# Hydrogen bond guidance and aromatic stacking drive liquid-liquid phase separation of intrinsically disordered histidine-rich peptides

**DOI:** 10.1038/s41467-019-13469-8

**Published:** 2019-11-29

**Authors:** Bartosz Gabryelczyk, Hao Cai, Xiangyan Shi, Yue Sun, Piet J. M. Swinkels, Stefan Salentinig, Konstantin Pervushin, Ali Miserez

**Affiliations:** 1 Center for Biomimetic Sensor Science, School of Materials Science and Engineering, Nanyang Technological University (NTU), 50 Nanyang Drive, Singapore, 637553 Singapore; 2 Department of Bioproducts and Biosystems, School of Chemical Engineering, Aalto University, Kemistintie 1, 02150 Espoo, Finland; 3 School of Physical and Mathematical Sciences, NTU, 21 Nanyang Link, Singapore, 637371 Singapore; 4 Physical Chemistry and Soft Matter, Wageningen University, 6708 WE Wageningen, Netherlands; 5 Laboratory for Biointerfaces, Department Materials Meet Life, EMPA, CH-9014 St-Gallen, Switzerland; 6 Department of Chemistry, University of Fribourg, Chemin du Musée 9, 1700 Fribourg, Switzerland; 7 School of Biological Sciences, NTU, 60 Nanyang Drive, Singapore, 637551 Singapore

**Keywords:** Biophysical chemistry, Biomaterials - proteins

## Abstract

Liquid-liquid phase separation (LLPS) of intrinsically disordered proteins (IDPs) is involved in both intracellular membraneless organelles and extracellular tissues. Despite growing understanding of LLPS, molecular-level mechanisms behind this process are still not fully established. Here, we use histidine-rich squid beak proteins (HBPs) as model IDPs to shed light on molecular interactions governing LLPS. We show that LLPS of HBPs is mediated though specific modular repeats. The morphology of separated phases (liquid-like versus hydrogels) correlates with the repeats’ hydrophobicity. Solution-state NMR indicates that LLPS is a multistep process initiated by deprotonation of histidine residues, followed by transient hydrogen bonding with tyrosine, and eventually by hydrophobic interactions. The microdroplets are stabilized by aromatic clustering of tyrosine residues exhibiting restricted molecular mobility in the nano-to-microsecond timescale according to solid-state NMR experiments. Our findings provide guidelines to rationally design pH-responsive peptides with LLPS ability for various applications, including bioinspired protocells and smart drug-delivery systems.

## Abstract (web summary)

Liquid-liquid phase separation (LLPS) of intrinsically disordered proteins plays an important part in the formation of extracellular biological materials. Here, the authors show that repeats of the peptide motif GHGLY are necessary for the LLPS of pH-responsive histidine-rich squid beak proteins.

## Introduction

Coacervation^1^ refers to the liquid–liquid phase separation (LLPS) of a homogeneous polymer solution into two distinct phases: a concentrated macromolecule-rich (or coacervate) phase and a dilute macromolecule-depleted phase^2^. Coacervation can occur between two oppositely charged polyelectrolytes (complex coacervation)^3^ or from self-association of a single polymer (self- or simple-coacervation)^4^. While coacervation studies were initiated in the field of biopolymeric colloids, in recent years LLPS has attracted considerable interest from life scientists^5,6^ with numerous studies showing its role in organizing biomolecules in living cells via formation of membraneless organelles^7–11^. Another less recognized but increasingly appreciated biological role of LLPS is associated with the assembly of extracellular, load-bearing structures^12^. A well-known example is tropoelastin, which undergoes self-coacervation upon secretion into the extracellular matrix where it self-assembles to form elastic fibers that provide strength and resilience to elastic tissues^4^. Coacervation has also been recognized to play a key role in natural bioadhesives secreted by marine invertebrates (for example the sandcastle tubeworm^3^ or mussels^13^) and to be involved in the formation of biological composite materials. In particular, we have recently identified and sequenced a family of proteins called histidine-rich beak proteins (HBPs) that are the main load-bearing component of the hard beak^14^ of the jumbo squid (*Dosidicus gigas*).

Recent studies of proteins involved in LLPS have revealed that such proteins usually belong to the family of intrinsically disordered proteins (IDPs) or contain intrinsically disordered regions (IDRs). IDPs that drive LLPS are typically characterized by conformational heterogeneity at equilibrium and by molecular motions that span timescales from ns to ms^15^. They usually also exhibit a low sequence complexity with a modular organization of their primary structure^6,16,17^. As a result, they lack a well-defined three-dimensional structure typical of globular proteins. It has been suggested that various intra- or intermolecular interactions are involved during LLPS of IDPs/IDRs, for example multivalent (cooperative), electrostatic, hydrophobic, or cation-π interactions^6,10^. Structure–function relationships of IDPs have primarily been obtained by site-directed mutagenesis, establishing the contributions of individual residues to the phase separation process^18–22^. However, molecular-scale interactions behind LLPS are still sparsely understood. A few NMR studies have provided direct experimental evidence linking protein sequence and structure with the ability to undergo LLPS. For example, a combined solution and solid-state NMR study on elastin-like peptides (ELPs) that exhibit LLPS through hydrophobic interactions triggered by temperature changes established a model by which the final biomaterial structure is self-assembled^23^. Solid-state NMR experiments have also been used to study the low complexity domain of the FUS^24^ and TDP-43 (ref. ^25^) RNA binding proteins, which undergo LLPS and in the pathological state may lead to the formation of insoluble fibril-like structures^26^.

A central characteristic of HBPs is the presence of repetitive regions of low complexity amino acid squence in their C-termini. Such molecular architecture is often found in extracellular IDPs with LLPS properties that are involved in the formation of biological structures with a load-bearing function^27^, for example tropoelastin^28^, resilin^29^, abductin^30^, and spider silk^31^. These repetitive regions are often enriched with hydrophobic residues that interact under specific conditions to trigger LLPS, which is a first step in the self-assembly process of the load-bearing tissue^12^. Besides hydrophobic residues, the repeats found in HBPs additionally contain a significant fraction of ionizable histidine (His) side chains. This feature is unique, and thus we selected HBP-1 as a model structural IDP to shed light on sequence motifs that govern LLPS as well as on intermolecular interactions stabilizing the coacervate phase.

Here, we combine mutagenesis studies with both solution- and solid-state NMR spectroscopy to investigate the self-coacervation process of HBPs. We systematically explore the HBP-1 sequence and identify that the motif repeat GHGLY drives LLPS. By studying various HBP-1-derived peptide sequences we find that when at least two copies of such repeats and a linker sequence are included, LLPS can be induced over a broader range of conditions (pH and salt concentration). Alternatively, at least four GHGLY tandem repeats must be present in order to trigger self-coacervation. Within this motif we show that His residues serve as a molecular switch: upon pH change, they first undergo deprotonation followed by hydrogen bonding with Tyr. Finally, using solution-, solid-state NMR, and small angle X-ray scattering (SAXS) we demonstrate that clustering of Tyr residues is critical to stabilize coacervate microdroplets.

## Results

### HBP-1 is structurally disordered in solution

**Fig. 1:**
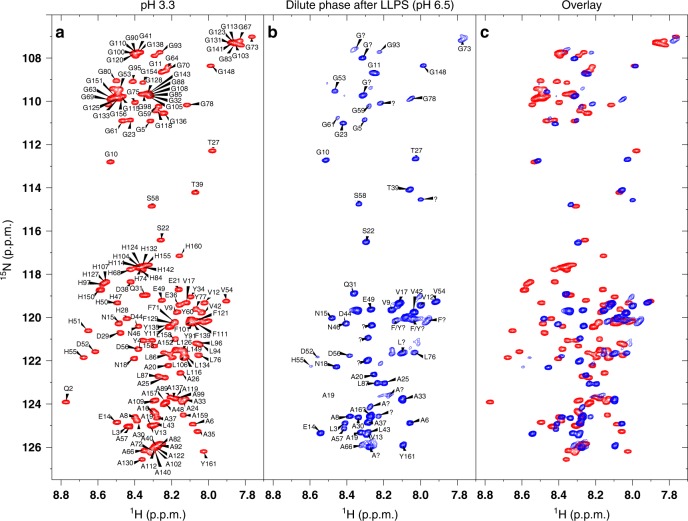
^1^H-^15^N-HSQC spectra of HBP-1 at different pH values. **a** HBP-1 in the initial solution state at pH 3.3. **b** Dilute phase after LLPS at pH 6.5 (after sedimentation of coacervate microdroplets). **c** Overlay of the two spectra. Spectra acquired at 298 K and a protein concentration of 2 mg mL^−1^ (130 μM).

HBP-1 possesses primary structure features characteristic of IDPs with LLPS properties (Supplementary Fig. 1). In a recent study, we showed using circular dichroism (CD) and SAXS that it has a disordered molecular structure in solution that transitions to a more ordered form in the coacervate state, and proposed that hydrophobic modular penta-repeats from the C-terminus are key to its self-coacervation process^32^. To verify these assumptions and investigate the structural features of the protein, we carried out a standard set of double- and triple-resonance NMR experiments with soluble recombinant HBP-1. As expected, NMR results indicated that the protein lacked a well-defined three-dimensional structure in solution: the ^1^H-^15^N heteronuclear single quantum coherence (HSQC) spectrum (Fig. 1a) showed narrow distribution of the cross-peaks, which is typically observed in IDPs with LLPS properties^25,33,34^. Analysis of the C_α_ and C_β_ chemical shifts of the assigned residues did not show significant deviations from random coil values, validating that the monomeric HBPs are uniformly disordered (Supplementary Fig. 2).

### C-terminal region of HBPs is involved in pH-dependent LLPS

LLPS of HBPs is triggered by changes in pH and ionic strength^32^. HBP-1 underwent LLPS at a minimal concentration of 20–30 µM in a narrow pH range 6.5–7.5, which is close to the proteins’ isoelectric point (predicted pI = 6.03) and could be broadened by increasing protein and salt concentration (phase diagrams presented in Supplementary Fig. 3). To precisely probe the residues involved in LLPS of HBP-1, we recorded a set of ^1^H-^15^N-HSQC spectra with a gradual increase of the pH from 3.3 (soluble state) (Fig. 1a) to 6.5 (at which point LLPS was initiated (Supplementary Fig. 4)). Finally, we measured the spectrum from the diluted phase after LLPS, when the coacervate microdroplets had sedimented (Fig. 1b). The overlay with the spectrum acquired in initial conditions (Fig. 1c) indicated the absence of resonances assigned to glycine (Gly), His, alanine (Ala), and leucine (Leu) residues located mainly in the C-terminal modular repetitive region, suggesting that these residues were involved in transient interactions that were absent at acidic pH. As a control we acquired a set of spectra at 75% lower concentration compared to the initial conditions (Supplementary Fig. 5) and at lower temperature (279 K vs. 298 K in initial conditions, Supplementary Fig. 6) to probe possible exchange between monomeric and oligomeric states or exchange with water molecules, respectively. For both experiments at pH 6.5, the intensity losses of the same cross-peaks were detected, confirming the specific involvement of these residues (located mostly in the modular repeats of HBP-1) during LLPS.

### Analysis of modular repeats driving phase separation of HBPs

**Fig. 2:**
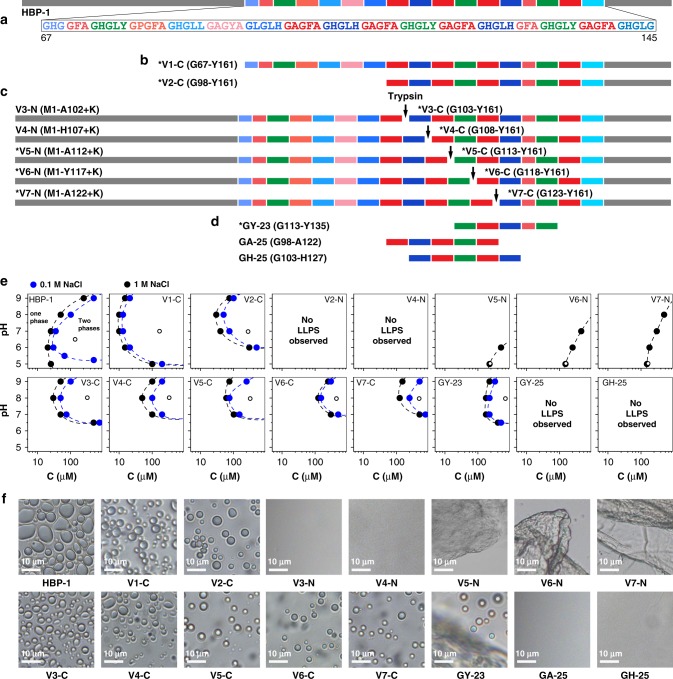
Analysis of LLPS properties of HBP-1 N- and C- terminal variants and peptides. **a** Amino acid sequence representation of HBP-1 protein. The repetitive region (G67–G145) is presented with modular repeats indicated with different color shades for motifs containing His (blue) or hydrophobic residues (red), and for the GHGLY motif (green). Non-repetitive N- and C-terminal regions are marked in gray. **b** C-terminal variants (V1-C containing the whole repetitive region, and V2-C truncated at position G98). **c** N- and C- variants obtained by trypsin cleavage. **d** Synthetic peptides. The same color marking was used for all peptides shown. Full amino acid sequences of all proteins and peptides are presented in Supplementary Figs. 1 and 7. Region of the HBP-1 sequence indicted in brackets. Variants that undergo LLPS marked with *. **e** Phase diagrams (protein or peptide concentration (C) on *x*-axis and pH on *y*-axis) at low (0.1 M) and high (1 M) salt concentrations, illustrating the conditions required to induce LLPS. As indicated in the upper-left panel (HBP-1), at low protein concentration only one phase is present (soluble protein). When LLPS occurs two phases co-exist, i.e. protein rich phase (coacervate microdroplets/hydrogel) and protein depleted diluted phase (the boundary lines between two phases are drawn as a guide for the eye). Black empty dots indicate pH and protein concentration at which optical micrographs presented in panel (**f**) were obtained. Source data are provided as a Source Data file. **f** Examples of optical micrographs taken after LLPS of all the variants and peptides described above and of HBP-1 (used as a control). Micrographs of V5-N, V6-N and V7-N represent hydrogels.

To study how the C-terminal modular domains’ arrangement influences self-coacervation of HBP-1, we designed a series of sequence variants (Fig. 2a–d, full sequences in Supplementary Fig. 7) and investigated their ability to phase separate at various pH and salt (NaCl) concentration using optical microscopy (Fig. 2e, f). First, we created a protein mutant lacking the first 66 amino acids but containing all modular repeats of the C-terminus (V1-C). This mutant underwent phase separation and formed coacervates at similar protein concentration and pH range compared to the full-length protein, confirming our hypothesis that C-terminal modular repeats are responsible for its phase separation behavior. Next, we studied a variant lacking the first 31 amino acids of the repetitive region (V2-C). This variant formed coacervates similarly to V1-C and HBP-1 wild type but required a slightly higher protein concentration (ca. 30 µM), indicating that the full length of the modular region was not required to induce phase separation.

To map out the minimal sequence length required for phase separation, we designed a series of HBP-1 mutants with various lengths of the repetitive region. The mutants were created by introducing a single Lys at different pre-selected locations, allowing to utilize trypsin cleavage to tune the length of the cleaved fragments following enzyme digestion as well as to obtain variants exhibiting different lengths of the repeating domains (Fig. 2c and Supplementary Fig. 7b).

We then analyzed the LLPS behavior of all variants as a function of protein concentration and pH, and at various salt concentrations and drew the phase diagrams shown in Fig. 2e. For N variants, LLPS occurred for V5-N to V7-N only at high salt concentrations. On the other hand, LLPS could not be induced for V3-N and V4-N at all tested conditions. We also observed that as peptide length increased, LLPS occurred over a broader range of conditions. Thus, for V7-N LLPS could be induced at pH as high as 8 provided the peptide concentration was at least 500 µM. For V6-N, the highest pH at which LLPS was observed was 7 (and a minimal peptide concentration of 400 µM), whereas for V5-N no LLPS occurred above pH 6. Correlating the results with the peptide design points out towards the importance of the GHGLY motif (marked in green in Fig. 2) and the peptide length. For the longer V6-N and V7-N peptides containing two GHGLY motifs, LLPS could be induced over a wider range of conditions, whereas for the shorter V3-N and V4-N variants containing only one copy of GHGLY, no LLPS was observed no matter the conditions. And for the intermediate length V5-N with one GHGLY motif, LLPS could be induced but only under narrow conditions. Moreover, the separated phases of the longer variants exhibited a different morphology compared to the full-length protein (Fig. 2f), forming dense hydrogel-like structures that did not disperse into the surrounding buffer. This behavior may be linked to the stronger hydrophobicity of V5-N to V7-N compared to other variants, which may favor hydrogel formation by hydrophobic interactions.

A similar trend was observed for the C-terminus variants. V3-C, which contained the longest section of the repetitive region, phase-separated at the lowest protein concentration (30 µM at pH 8) and in the broadest pH range among all tested variants. On the other hand, the shorter Vx-C variants exhibited LLPS under a narrower range of conditions and required higher protein concentrations.

To further assess the role of the GHGLY motif, we compared the coacervation ability of the HBP-1 derived GY-23 peptide (containing two GHGLY copies)^32^ with two other synthetic peptides made of very similar fragments of HBP-1 repeats (GA-25 and GH-25), but harboring only one GHGLY motif (Fig. 2d and Supplementary Fig. 7c). Only GY-23 phase-separated, forming coacervate microdroplets suspended in solution as well as a dense hydrogel-like structure (condensed, solid-like coacervates, Fig. 2f). In contrast, GA-25 and GH-25 remained in solution in all tested buffer conditions (Fig. 2f). We note that sequence motifs similar to GHGLY are also present in the C-terminal of HBP-2 protein, which contains seven copies of the GHGxY motif (where x can be Val, Pro, Leu) arranged in tandem (Supplementary Fig. 8). A peptide (HBP-2-pep) composed of five copies of GHGxY was previously shown to phase separate and form coacervates in the same way as the full-length protein^14^.

**Fig. 3:**
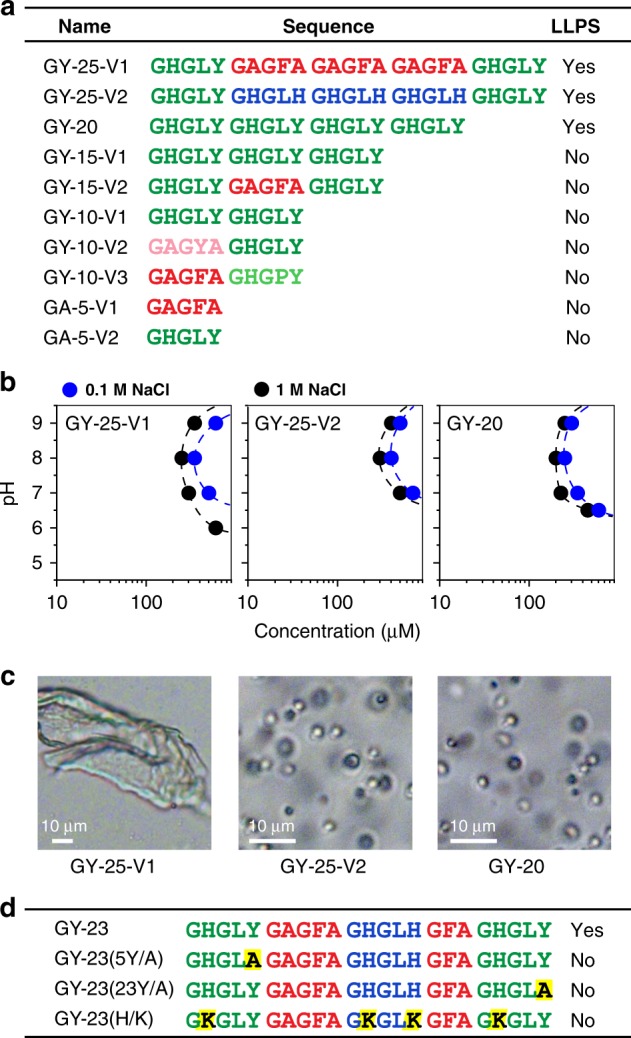
LLPS properties of HBP-1 and -2 derived peptides. **a** Sequences and their ability to undergo LLPS. **b** Phase diagrams of the peptides that exhibited LLPS properties. Source data are provided as a Source Data file. **c** Sample morphology after LLPS by optical microscopy (left micrograph: hydrogel; middle and right micrographs: microdroplets). **d** Site-directed mutants of GY-23 peptide and their LLPS ability. Color marking of HBP-1 modular repeats is identical to the color-coding described in Fig. 2. All samples were tested in the same conditions in various pH values and salt concentrations.

In order to confirm the central role of GHGxY motifs on LLPS of HBP-2, we utilized trypsin cleavage to obtain shorter fragments of HBP-2 and tested their ability to phase separate. Since the protein possesses only two trypsin recognition sites at positions R81 and R172, we obtained the N-terminal (M1-R81) fragment that lacked the modular repeats, the C-terminal (A82-R172) containing the whole repetitive region, and a short G173-Y175 peptide that was discarded (Supplementary Fig. 8b). As expected only the C-terminal fragment phase-separated into coacervates (Supplementary Fig. 8c). Next, we designed a series of short peptides containing different arrangement of repetitive units present in HBP-1 and HBP-2 (Fig. 3a) and analyzed their phase separation behavior in the same way as for HBP-1 variants (Fig. 3b, c). Phase separation was observed for all 25-mer peptides containing two GHGLY motifs flanking the central region composed of three copies of the GAGFA or GHGLH sequences, as well as for a 20-mer peptide (GY-20) made of four copies of GHGLY motif arranged as tandem repeats. In contrast, no phase separation was observed when the peptide length was reduced to 15 amino acids, for example when three copies of the GHGLY motif were arranged in tandem (GY-15-V1) or when the GAGFA motif was flanked by GHGLY (GY-15-V2). Similarly, no phase separation was observed for decapeptides composed of one or two GHGxY motifs or for pentapeptides GHGLY or GAGFA, respectively. Moreover, peptides with LLPS ability exhibited various rheological characteristics of the separated phase. GY-25-V1 peptide containing three copies of hydrophobic GAGFA motif phase-separated into a dense and compact hydrogel. On the other hand, GY-25-V2 and GY-20 peptides composed of less hydrophobic, His-rich motifs, only formed microdroplets (Fig. 3c), while GY-23 peptide containing both types of motifs separated into microdroplets as well as hydrogel-like condensed coacervates (Fig. 2f).

Taken together these results indicate that when at least two copies of the GHGLY motif are present in the tandem repeats, the phase separation ability is greatly enhanced. However, this condition is not sufficient and GHGLY copies must additionally be separated by a spacer composed of at least three copies of GAGFA or GHGLH motifs, or a combination of GAGFA/GFA and GHGLH motifs. Alternatively, the peptide must contain at least four tandem repeats of GHGLY motif to phase separate. To corroborate the role of Tyr in phase separation, we prepared two GY-23 variants in which one of two Tyr was substituted with Ala (Fig. 3d). Phase separation did not occur in both cases in all tested conditions, suggesting that it is critical to have two Tyr residues to drive phase separation. Finally, we investigated the LLPS ability of the GY23(H/K) mutant in which all His were substituted with Lys. This peptide did not undergo LLPS at all tested conditions, showing that the role of His residues in HBP peptides is not limited to shifting the net charge of the peptides as the pH changes. Instead, this result indicates that histidine residues are involved in additional interactions driving the LLPS process, as discussed below.

### Molecular interactions initiating LLPS

**Fig. 4:**
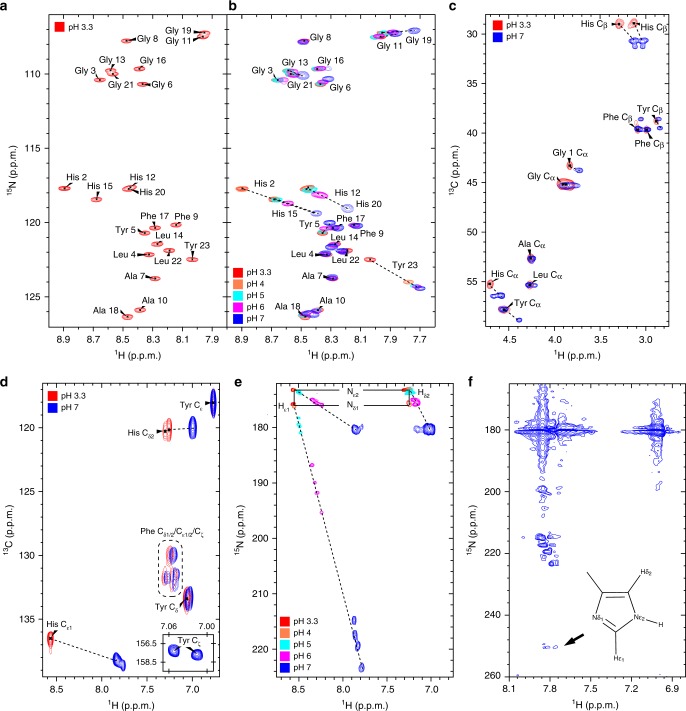
NMR spectra of GY-23 peptide at different pH values (cross-peak trajectories marked with dashed lines). **a** ^1^H-^15^N-HMQC spectrum at initial conditions of pH 3.3. **b** Overlay of ^1^H-^15^N-HMQC spectra acquired between pH 3.3 and 7 (pH 7: initiation of LLPS). **c**, **d** Overlay of ^1^H-^13^C-HSQC spectra of aliphatic (**c**) and aromatic (**d**) side chains at pH 3.3 and 7. The inset shows Tyr ^1^H_δ_-^13^C_ζ_ cross-peaks at pH 7. **e** Overlay of long-range ^1^H-^15^N-HMQC spectra of His side chains. The resonance assignments in the protonated state (pH 3.3) are indicated. **f** Long-range ^1^H-^15^N-HMQC spectrum at pH 7 acquired within 5 min after pH adjustment showing transient stabilization of His ε-tautomer with characteristic resonance at ca. 250 ppm marked with the arrow. In the spectrum acquired after 30 min of pH adjustment, this cross-peak was significantly attenuated (Supplementary Fig. 11). Spectra acquired at 298 K and peptide concentration of 1.5 mM. The trajectories (chemical shift values vs. pH) of ^13^C atoms of Tyr as well as ^13^C and ^15^N of His are provided in Supplementary Fig. 12.

To assess the role of Tyr residues and identify the detailed molecular interactions triggering and stabilizing LLPS, we carried out NMR spectroscopy studies. First, we acquired the ^1^H-^15^N-HMQC spectrum in solution as well as a set of triple-resonance NMR spectra for peptide backbone assignment of soluble GY-23 at pH 3.3. The ^1^H-^15^N-HMQC spectrum yielded well-resolved peaks that could be fully assigned based on the carbon chemical shifts values obtained from the 3D experiments (Fig. 4a). Observed C_α_ and C_β_ chemical shifts showed no significant differences from the average values of random coil structures (Supplementary Fig. 9), confirming that the peptide displayed no propensity towards a specific secondary structure.

Next, we titrated the pH of the peptide solution and monitored changes in the ^1^H-^15^N-HMQC (Fig. 4b). We did not observe major variations in the peak distribution and relative intensity at pH 4–6 (Supplementary Fig. 10) compared to the initial state (pH 3.3, Fig. 4a), since in these conditions the peptide remained fully soluble (except of the Tyr 23 cross-peak that showed a significant shift when the pH changed from 3.3 to 4 caused by deprotonation of the C-terminal carboxyl group). However, close to the LLPS point (pH 6–7), there was a clear shift and decrease in the relative intensity of all cross-peaks assigned to His residues (Supplementary Fig. 10), as well shifts of all Gly peaks flanking them. In addition, we observed shifts of the cross-peak assigned to Tyr 5 (Fig. 4b).

These results indicated that His and Tyr residues are involved in initiating LLPS. To investigate their role in the initial steps of aggregation, we carried out pH titration experiments on GY-23, where we recorded ^1^H-^13^C-HSQC spectra of aliphatic (Fig. 4c) and aromatic (Fig. 4d) side chains of all residues, as well as the long-range ^1^H-^15^N-HMQC spectra to monitor the protonation state of nitrogen atoms in the imidazole ring of His (Fig. 4e, f and Supplementary Fig. 11). Increasing the pH led to gradual changes of the chemical shifts of His ^13^C_α_ and ^13^C_β_ atoms (Fig. 4c), as well as of ^13^C_δ_ and ^13^C_ε_ atoms of the imidazole ring (Fig. 4d). In addition, when the pH was raised from 3 to 4 the cross-peaks assigned to ^13^C_α_ and ^13^C_β_ of the C-terminal residue Tyr 23 significantly shifted in the ^1^H and ^13^C dimensions, suggesting that the shift is caused by deprotonation of the C-terminal carboxylic group. We also observed a major shift of the ^13^C_α_ cross-peak assigned to Gly 1 (Fig. 4c).

Aromatic ^1^H-^13^C-HSQC spectra showed (Fig. 4d) that increasing pH results in gradual shifts of the cross-peaks assigned to ^13^C_δ2_ and ^13^C_ε1_ of His residues, caused by deprotonation of the imidazole ring. Resonances assigned to Phe remained unaffected by change of pH between 3.3 and 6.0 but when pH increased to 7.0 we detected a shift of all Phe ^1^H resonances (Fig. 4d). Tyr resonances showed similar trend, except ^13^C_ζ_ that started to split at pH 5.0. With further increase of the pH we observed the presence of two distinct cross-peaks ^13^C_ζ_ atoms of Tyr 5 and Tyr 23 (Fig. 4d, inset). In addition we also observed a split of Tyr ^13^C_δ_ resonances into two cross-peaks when pH increased from 6.0 to 7.0. Figure 4e shows changes in chemical shifts of ^15^N atoms of His imidazole ring during pH titration. At pH 3.3 and 4 all His were fully protonated as indicated by characteristic the ^15^N_ε2_ and ^15^N_δ1_ chemical shifts, i.e. 173 ppm and 176 ppm, respectively^35^. Increasing pH from 4 to 7 led to a gradual deprotonation of the imidazole rings of all His, resulting in the co-existence of the protonated state with two tautomeric forms of the imidazole ring. Critically, we observed that immediately after raising the pH from 6 to 7, only one of four His residues showed transient stabilization of its ε tautomer state since the ^15^N_δ1_ cross-peak also appeared at 250 ppm^36^ within 5 min after pH adjustment (Fig. 4f). However, the cross-peak intensity was significantly reduced 30 min after pH adjustment (Supplementary Fig. 11a, b), indicating that only one His residue underwent transient stabilization of the tautomeric state, which was likely caused by hydrogen bonding. Since between pH 5 and 7 the chemical shifts of Tyr ^13^C_ζ_ atoms split into two distinct shifts (Fig. 4d, inset), this suggests that hydrogen bond interaction is taking place between the hydroxyl group of Tyr (donor) and ^15^N_δ1_ of His (acceptor), which may be the first step in the oligomerization cascade. Detailed analysis of chemical shift trajectories presented in Supplementary Fig. 12 further confirm these observations. Moreover, we carried out 3D ^15^N- and ^13^C-NOESY experiments with long mixing times and did not observe NOEs between His and Tyr, further supporting the transient character of the Tyr/His interactions.

### GY-23 peptide shows partially ordered structure after LLPS

**Fig. 5:**
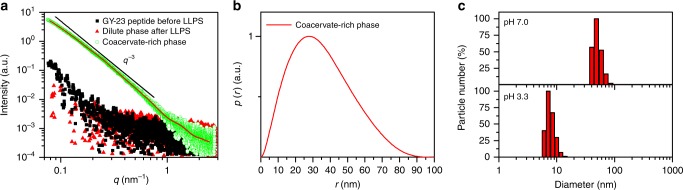
SAXS and DLS of GY-23 peptide. **a** SAXS experimental curves of the peptide before and after coacervation. After LLPS, the dilute and coacervate-rich phases were measured following a centrifugation step. The *q*^-3^ power-law region of the scattering data is highlighted, with the black line as a guide for the eye. The calculated fit for the peptide assemblies from the IFT method is also presented as a continuous red line. **b** Corresponding *p*(*r*) profile calculated from the SAXS data in (**a**) using Supplementary Eq. 2. **c** Hydrodynamic diameter (*D*_H_) measured by DLS of GY-23 before (pH 3.3) and after (pH 7.0) coacervation. Correlation functions showing the ‘raw’ data are presented in Supplementary Fig. 14. Source data are provided as a Source Data file.

Although IDPs do not exhibit well-defined tertiary structures, there are evidences that coacervate microdroplets of IDPs contain short-range order^10^. To further study the coacervation at the nanostructural level and assess whether GY-23 coacervate microdroplets exhibited such internal ordering, we investigated their structural features using SAXS. Scattering profiles of GY-23 in acetic acid (pH 3.3) before LLPS and in the coacervation buffer (pH 7.0) after LLPS (both the coacervate and the coexisting dilute phases) are presented in Fig. 5a and were very distinct from each other. The scattering intensity of GY-23 in acetic acid and of the dilute phase after centrifugation had a very low signal-to-noise ratio. Nevertheless, for GY-23 in acetic acid, a weak low-*q* upturn with an indication of a broad correlation peak between 0.3 and 2 nm^-1^ was observed, which may be attributed to nanometer-sized peptide oligomers. Dynamic light scattering (DLS) analysis of the peptide in acetic acid (Fig. 5c) indicated the presence of structures with a hydrodynamic diameter (*D*_H_) of ca. 8 nm, corroborating the presence of small oligomeric units (assuming *D*_H_ on the order of 4–8 nm for the 23 residue-long monomeric peptide). As expected, *D*_H_ increased drastically to around 50 nm at pH 7.0 due to initiation of LLPS.

In contrast, the scattering profile of GY-23 in the peptide-rich phase (Fig. 5a) indicated the presence of much larger peptide aggregates typical for coacervate microdroplets with overall dimensions that exceeded the resolution limit of the SAXS set-up. An indication of a broad correlation peak in the *q*-region of ~1.5 nm^-1^ suggested structural features from peptide self-assemblies within the coacervate microdroplets. The low signal-to-noise ratio in this *q*-region makes it difficult to analyze this feature in detail (however, this correlation peak was confirmed using a more intense synchrotron X-ray source, Supplementary Fig. 13). At *q* < 1 nm^-1^, on the other hand, the scattering curve showed an approximate power-law dependence over at least an order of magnitude in the *q*-range, indicating fractal scattering from the dense peptide assemblies within the coacervate phase.

To further investigate the internal structure of coacervate microdroplets, the pair distance distribution function *p*(*r*) was calculated from the SAXS curve using the indirect Fourier transformation (IFT) method (Fig. 5b). The *p*(r) function reflected large peptide aggregates in the microdroplets with dimensions well-beyond the resolution limit of the SAXS set-up in this study (around 50 nm in real space). Hence, the *p*(r) was mathematically forced to 0 at *r* around 100 nm, but this does not represent the overall dimension of the coacervate microdroplets. The analysis of the corresponding SAXS data of the coacervate droplets in buffer at a higher signal-to-noise ratio, recorded at the synchrotron, is presented in the Supplementary Fig. 13. The results indicated that the coacervates microdroplets contained nanostructural features of ca. 2 nm. These features are most likely attributed to oligomeric peptides forming the internal domain structures of the coacervate microdroplets.

### Analysis of tyrosine–tyrosine interactions by ssNMR

**Fig. 6:**
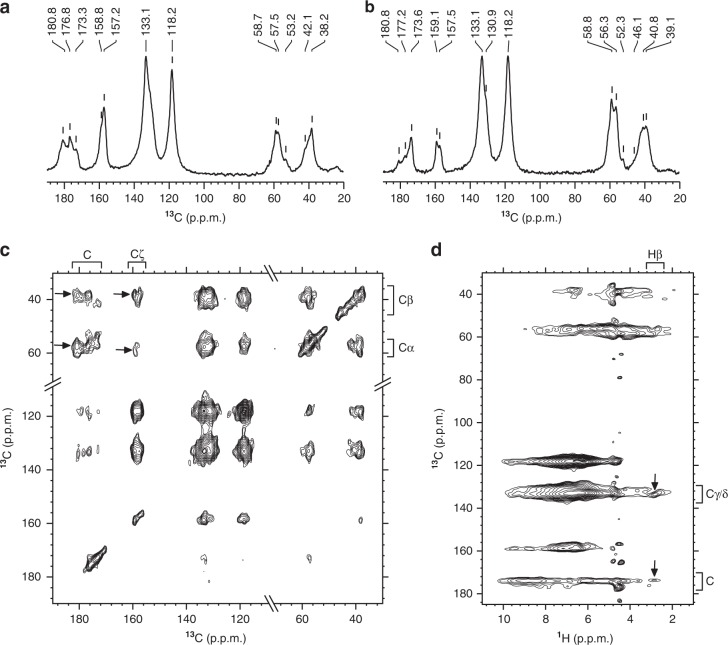
Characterization of molecular interactions driving LLPS of GY-23 peptide by ssNMR. Spectra of ^13^C-selectively Tyr 5 and Tyr 23 labeled GY-23: (**a**) directly observed carbon, (**b**) ^1^H-^13^C cross-polarization (CP)-based with carbon detection, (**c**) DARR (100 ms mixing time), and (**d**) ^1^H-^13^C HETCOR (100 μs mixing time). Examples of correlations indicating Tyr–Tyr interactions are marked with arrows.

Since site-directed mutagenesis experiments suggested a critical role of Tyr residues, we synthesized GY-23 containing uniformly labeled (^13^C and ^15^N) Tyr residues (Tyr 5 and Tyr 23) and analyzed possible Tyr–Tyr interactions in the condensed, solid-like phase by solid-state NMR. Figure 6a, b show a comparison between the one-dimensional direct- and ^1^H-^13^C cross-polarization (CP)-based carbon spectra. Both spectra contain relatively broad lines, indicating that Tyr residues were present in heterogeneous conformational environments since multiple peaks for each Tyr carbon were observed. For example, ^13^C_α_ resonances at 53.2 ppm, 57.5 ppm, 58.7 ppm, and carbonyl ^13^C at 173.3 ppm, 176.8 ppm, 180.8 ppm, respectively, were detected. The presence of strong signals in the CP-based spectrum indicated that most of Tyr moieties were locked in the rigid structure with high dipolar order. No extra sharp peak was observed in the direct-polarization ^13^C spectrum compared with the CP-based spectrum, indicating the absence of highly flexible Tyr residues and further supporting that Tyr residues were rigidly locked. This is further confirmed by a control experiment in which we recorded the same set of spectra from the sample at pH 6 and 7 (Supplementary Fig. 15). At pH 6, the intensity of the CP signal decreased compared to pH 7, revealing an increased mobility of Tyr residues. Moreover, the directly detected ^13^C spectrum displayed sharper peaks at pH 6, thus corroborating the increased mobility of Tyr at low pH.

The two-dimensional ^13^C–^13^C dipolar assisted rotational resonance (DARR) spectrum (Fig. 6c) showed correlations between the two Tyr residues of the peptide, suggesting that they interacted with each other. Moreover, the DARR data clearly indicated that Tyr residues were in heterogeneous chemical environments, implying clustering of Tyr residues close to each other. Tyr–Tyr direct interactions were also corroborated by the heteronuclear correlation (HETCOR) spectrum (Fig. 6d), which shows correlations between aliphatic and aromatic carbon atoms of Tyr attributed to the stacked clustering of two or more Tyr side groups.

## Discussion

**Fig. 7:**
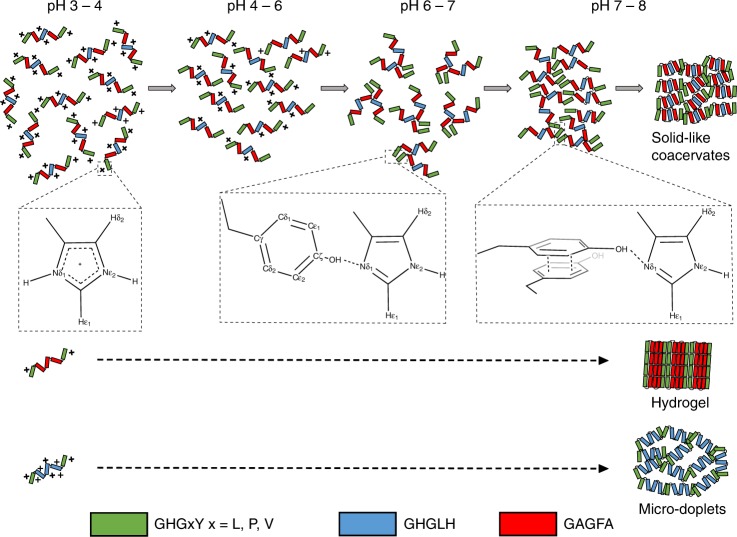
Proposed model of pH-dependent LLPS of HBP-derived peptides. At pH 3–4 His residues are protonated, and the peptides form soluble oligomeric units due to electrostatic repulsion between positively charged His side chains. At pH 4–6 gradual deprotonation of His residues occurs, repulsive forces are weaker but still strong enough to keep the peptide oligomers soluble. At pH 6–7 transient interactions take place between His and Tyr residues located within GHGxY repeats (marked in green) leading to specific peptide-peptide interactions that act as nuclei for LLPS. Further increase of pH above 7 leads to Tyr–Tyr intermolecular stacking and intra-molecular interaction of hydrophobic residues that all together trigger LLPS and the formation of microdroplets. If the central domain of the peptide is enriched with the hydrophobic motif GAGFA (marked in red) or with the His-rich motif GHGLH (marked in blue), LLPS is driven by the same sequence of molecular events but eventually leads to the formation of either a hydrogel or coacervate micro-droplets, respectively.

There has been growing recognition that LLPS is involved inside cells *via* membraneless organelles^6–8,10,11,26^ as well as in the processing of extracellular load-bearing structures and bioadhesives of various organisms^3,4,12–14,32,37–39^. However, sequence motifs and associated inter- and intra-molecular interactions driving phase separation remain sparsely understood. This study enhances our understanding of LLPS phenomena both at the sequence and molecular levels. Our findings show that phase separation of HBPs is mediated through specific GHGxY modular repeats that must be arranged in a specific configuration. Our results also show that the morphology and rheology of separated phases can be tuned from coacervate microdroplets to hydrogels by incorporating hydrophobic GAGFA repeats into a peptide sequence. Based on solution-state NMR measurements, LLPS of HBPs is a multistep process initially triggered by deprotonation of His residues upon pH increase, followed by stabilization of His ε tautomeric state by transient hydrogen bonding with OH group of Tyr residues. We propose that these events eventually promote hydrophobic intermolecular interactions largely controlled by Tyr residues, as well as hydrophobic collapse of the peptides’ central domains as schematically illustrated in Fig. 7. Investigations of the GY-23 coacervate phase by SAXS and solid-state NMR indicated that it possesses partial internal ordering in the nanometer range that is stabilized by aromatic stacking and clustering of Tyr residues. These findings concur with earlier biophysical studies on the full length HBPs showing that a certain degree of protein folding is achieved in the coacervate state^32^.

There are a few reports providing a full picture of molecular events leading to LLPS of IDPs^23–25^. One relevant study by Reichheld et al.^23^ showed that self-coacervation of ELPs is an entropy-driven mechanism mediated by transient interactions between the highly dynamic and disordered hydrophobic domains of ELPs. Hydrophobic interactions led to gradual exclusion of water and salt molecules, eventually allowing chemical crosslinking of ELP monomers to form an elastic network. According to our recent studies, the mechanism of self-coacervation of HBPs is also entropy-driven and involves hydrophobic interactions of repetitive domains^32^. In contrast to ELPs, those interactions are triggered by deprotonation of His residues, followed by hydrophobic interactions leading to gradual condensation of HBP coacervates. This is in line with our model of squid beak processing in vivo, which assumes that HBP coacervates infiltrate, condensate, and dehydrate a chitin nanofiber scaffold present in the squid beak and finally undergo chemical crosslinking^14,40^. Therefore, the partial ordering of the HBP coacervates that we observed by SAXS and solid-state NMR may be an intermediate step before the final crosslinking taking place in vivo. Moreover, the formation of solid materials through condensation processes (i.e. transitions from liquid to solid state) of macromolecular assemblies during LLPS is not typical only for extracellular structures. It is a common process observed also inside cells. For example, a heterochromatin protein 1α^41^ can undergo time dependent condensation into a gel, while RNA binding proteins^42^ or stress granule proteins^43^ can form insoluble aggregates often related with pathological states.

There is increasing evidence that π–π stacking is critical to drive LLPS and stabilize phase-separated structures, for example in the mitotic spindle regulatory protein BuGZ^19^, the nuclear pore protein Nsp1 (ref. ^22^), or FUS^44,45^. Another model of LLPS that involves aromatic residues is based on π-cation interactions between positively charged residues (Arg or Lys) and aromatic moieties of Phe or Tyr^18,46,47^. Our study shows that Tyr–Tyr interactions are critical to stabilize the biopolymer-rich phase after phase separation, but that they must first be activated through interactions with His side groups in a pH-dependent mechanism. To the best of our knowledge, this multistep interaction mechanism has previously not been reported in IDPs and provides a better understanding of pH-responsive LLPS.

Our findings also have implications in the design of stimuli-responsive protein carriers for various therapeutic treatments. Indeed, the family of GHGxY-containing peptides described in this study expands our molecular toolbox of peptides-forming coacervates for therapeutics delivery^48–51^ beyond the classical ELPs^52,53^, in particular offering the added advantages to design and tune pH-responsive carriers de novo as well as the ability to package hydrophilic drugs inside the coacervate microdroplets.

## Methods

### Assessment of LLPS properties

LLPS properties of HBP-1 protein, its variants and HBP peptides at different buffer conditions (Figs. 2–3 and Supplementary Fig. 3, list of the buffers presented in Supplementary Table 1) were assessed using the method described by Tan et al.^14^. Briefly, protein/peptide stock solution (in 10 mM acetic acid, pH 3.3) was added to a buffer solution in a volume ratio 1:5 (protein/peptide stock:buffer). The mixture was then pipetted onto a microscopy glass slide and imaged using an optical microscope.

### Optical microscopy

The phase separation behavior of protein variants and peptides was studied using a Zeiss Axio Scope A1 microscope (Carl Zeiss Pte Ltd., Germany) in the reflection mode, with differential interference contrast (DIC) filters. Images were taken with an AxioCam MRc 5 camera under the control of AxioVision software.

### Solution-state NMR spectroscopy

Lyophilized HBP-1 protein or GY-23 peptide samples were dissolved in 10 mM acetic acid (pH 3.3) containing 10% D_2_O and 0.2 mM DSS prior the NMR experiments. 0.5 M NaOH was used for pH adjustment during pH titration experiments.

For HBP-1 protein backbone assignment, three-dimensional BEST-TROSY HNCO, HNCA, HN(CO)CA, HNCACB, HN(CO)CACB, HN(CA)CO experiments^54^ were recorded on a 700 MHz Bruker Advance III NMR spectrometer equipped with 5 mm z-gradient TXI cryoprobe operating at 298 K. The spectra were acquired using non-uniform sampling (NUS) with 30% amount of sparse sampling. Processing of the NUS spectra was performed using MDDNMR program^55^ implemented in TopSpin 3.5 (Bruker) software. Backbone assignment was carried out using CARA software (http://cara.nmr.ch/). ^1^H-^15^N-HMQC spectra at different pHs were acquired using SOFAST-HMQC pulse program^56^ on an 800 MHz Bruker Advance III NMR instrument equipped with 5 mm QCI H/P/C/N solution cryoprobe, at 298 K or 279 K.

Data for GY-23 backbone assignment were collected on the 800 MHz spectrometer. The same set of BEST-TROSY experiments (as for HBP-1 protein, expect of HN(CO)CACB) were recorded utilizing NUS with 10–30% amount of sparse sampling. Processing of the data and backbone assignment was performed as described above. Experiments during pH titration: ^1^H-^15^N-HMQC, ^1^H-^13^C-HSQC, and long-range ^1^H-^15^N-HMQC spectra of His side chains were acquired using standard pulse programs from the TopSpin 3.5 repository on the 700 MHz spectrometer. ^15^N- and ^13^C-HSQC-NOESY with 500 ms mixing time were acquired on the 600 MHz Bruker Advance III spectrometer equipped with 5 mm z-gradient TCI cryoprobe, at 298 K.

### SAXS

Sample was prepared by dissolving 5.0 mg of lyophilized GY-23 peptide in 100 µL of 10 mM acetic acid (pH 3.3). Coacervation was induced by mixing of the peptide stock with the coacervation buffer (50 mM Tris-HCl, pH 7.0 buffer, containing 1 M NaCl) in 1 to 5 volume ratio. Coacervate-rich phase was collected by centrifugation (13,000*g* for 5 min at 25 °C) and transferred into a 1.5 mm quartz capillary together with some supernatant to avoid drying. The position of the capillary was then specifically aligned to hit the coacervate-rich phase.

SAXS measurements were performed on a Bruker Nanostar U (Bruker AXS, Karlsruhe, Germany) connected to a sealed-tube Cu anode X-ray source operating at 50 kV and 600 μA (Incoatec IμSCu, Geest-hacht, Germany). A Göbel mirror was used to convert the divergent polychromatic X-ray beam into a focused beam of monochromatic Cu Kα radiation (λ = 0.154 nm). The beam size was 0.3 mm. A sample to detector distance of 1077 mm gave the q-range 0.07 < *q* < 2.9 nm^−1^. The 2D SAXS patterns were acquired within 1 h using a VÅNTEC-2000 detector (Bruker AXS, Karlsruhe, Germany) with an active area of 140 × 140 mm^2^ and a pixel size of 68 μm.

The samples were measured in 1.5 mm quartz capillaries. The scattering curves were plotted as a function of intensity, *I* vs. *q*. Scattering from the corresponding buffer was subtracted as background from all samples.

### Dynamic light scattering

DLS measurements were performed on ZetaPALs (Brookhaven Instruments Corporation) equipped with a 35 mW red diode laser (640 nm wavelength). The scattering angle was set to be 90° and each sample was measure 5 times.

Sample was prepared by dissolving of 1 mg of the GY-23 peptide in 100 µl of 10 mM acetic acid. Coacervation was induced by rising pH to 7.0 (adjusted with 1 M NaOH) and addition of salt (NaCl).

### Solid-state NMR spectroscopy

HBP-1 and GY-23 peptide coacervates were loaded directly into 1.9 mm MAS rotor by ultracentrifugation (100,000 *g*, 30 min, 20 °C) using spiNpack (Giotto Biotech, Italy) rotor packing device. NMR data were collected on a 600 MHz Bruker Advance III instrument equipped with a 1.9 mm MAS probe operating in HX double resonance mode. One-dimensional (1D) ^1^H–^13^C cross-polarization (CP), ^13^C direct-polarization (DP) and 2D ^13^C–^13^C dipolar assisted rational resonance (DARR) experiments were performed with the MAS spinning frequency set at 18 kHz and the variable temperature set at 2 °C. The actual sample temperature was 10 °C based on the external calibration with ethylene-glycol^57^. Chemical shifts were referenced using the DSS scale with adamantane as a secondary standard for ^13^C^58^ (downfield signal at 40.48 ppm) and were calculated indirectly for ^1^H. The ^1^H→^13^C CP transfer was achieved by using 56 kHz ^13^C and 81 kHz (maximum power) ^1^H spin-lock rf fields with a 90–100% linear ramp applied on the ^1^H channel and a contact time of 250 μs. 80 kHz SPINAL-64 ^1^H decoupling was implemented during data acquisition. The recycle delays were 1.5 s and 5 s in the 1D CP and DP experiments, respectively, and the acquisition time was 19.1 ms in both experiments. Additional parameters of the 2D ^13^C–^13^C DARR experiment included 1.5 s recycle delay, 72115.4 Hz sweep width and 14.2 ms acquisition time in the direct dimension, 36000 Hz sweep width and 7.1 ms acquisition time in the indirect dimension and 100 ms DARR mixing time. A dipolar based 2D ^1^H–^13^C heteronuclear correlation (HETCOR) experiment was conducted with 35 kHz MAS rate. The variable temperature was maintained at 15 °C corresponding to 13 °C actual sample temperature. 86 kHz ^1^H and 50 kHz (maximum power) ^13^C spin-lock rf fields with a 90–100% linear ramp applied on the ^13^C channel were implemented for the ^1^H→^13^C and ^13^C→^1^H CP transfers and the contact time was 100 μs. Suppression of water signal was achieved by implementing the MISSISSIPPI scheme without the homospoil gradient^58^. Additional parameters of the 2D ^1^H–^13^C HETCOR experiment included 1.5 s recycle delay, 34722.2 Hz sweep width and 11.1 ms acquisition time in the direct dimension, 35000 Hz sweep width and 7.3 ms acquisition time in the indirect dimension, 10 kHz XiX ^1^H decoupling during ^13^C chemical shift evolution period and 10 kHz WALTZ-16 ^13^C decoupling during ^1^H acquisition time.

## Supplementary information

Supplementary Information

## Data Availability

The authors declare that all relevant data supporting the findings of this study are available within the paper and its Supplementary Information files. The source data underlying Figs. 2e, 3b, 5a–c, and Supplementary Figs. 1, 2, 3, 9, 10, 12, 13a, b, and 14a, b are provided as a Source Data file. Additional data are available from the corresponding author on request.

## Source data

Source Data

## References

[1] Bungenberg de Jong HG, Kruyt HR (1929). Coacervation (partial miscibility in colloid systems). Proc. Acad. Sci. Amst..

[2] De Kruif CG, Weinbreck F, De Vries R (2004). Complex coacervation of proteins and anionic polysaccharides. Curr. Opin. Colloid Interface Sci..

[3] Zhao H, Sun C, Stewart RJ, Waite JH (2005). Cement proteins of the tube-building polychaete *Phragmatopoma californica*. J. Biol. Chem..

[4] Yeo GC, Keeley FW, Weiss AS (2011). Coacervation of tropoelastin. Adv. Colloid Interface Sci..

[5] Dzuricky M, Roberts S, Chilkoti A (2018). Convergence of artificial protein polymers and intrinsically disordered proteins. Biochemistry.

[6] Brangwynne CP, Tompa P, Pappu RV (2015). Polymer physics of intracellular phase transitions. Nat. Phys..

[7] Mitrea DM, Kriwacki RW (2016). Phase separation in biology; functional organization of a higher order. Cell Commun. Signal.

[8] Holehouse AS, Pappu RV (2018). Functional implications of intracellular phase transitions. Biochemistry.

[9] Boeynaems S (2018). Protein phase separation: a new phase in cell biology. Trends Cell Biol..

[10] Banani SF, Lee HO, Hyman AA, Rosen MK (2017). Biomolecular condensates: Organizers of cellular biochemistry. Nat. Rev. Mol. Cell Biol..

[11] Hyman AA, Weber CA, Jülicher F (2014). Liquid-liquid phase separation in biology. Annu. Rev. Cell Dev. Biol..

[12] Muiznieks LD, Sharpe S, Pomès R, Keeley FW (2018). Role of liquid–liquid phase separation in assembly of elastin and other extracellular matrix proteins. J. Mol. Biol..

[13] Wei W (2014). A mussel-derived one component adhesive coacervate. Acta Biomater..

[14] Tan Y (2015). Infiltration of chitin by protein coacervates defines the squid beak mechanical gradient. Nat. Chem. Biol..

[15] Salvi, N., Abyzov, A. & Blackledge, M. Analytical description of NMR relaxation highlights correlated dynamics in intrinsically disordered proteins.* Angew. Chem. Int. Ed. Engl.* **56**, 14020–14024 (2017). 10.1002/anie.201706740 28834051

[16] Van Der Lee R (2014). Classification of intrinsically disordered regions and proteins. Chem. Rev..

[17] Uversky VN (2017). Protein intrinsic disorder-based liquid–liquid phase transitions in biological systems: Complex coacervates and membrane-less organelles. Adv. Colloid Interface Sci..

[18] Nott TJ (2015). Phase transition of a disordered nuage protein generates environmentally responsive membraneless organelles. Mol. Cell.

[19] Jiang H (2015). Phase transition of spindle-associated protein regulate spindle apparatus assembly. Cell.

[20] Lin Y, Currie SL, Rosen MK (2017). Intrinsically disordered sequences enable modulation of protein phase separation through distributed tyrosine motifs. J. Biol. Chem..

[21] Wang J (2018). A molecular grammar governing the driving forces for phase separation of prion-like RNA binding proteins. Cell.

[22] Frey S, Richter RP, Görlich D (2006). FG-rich repeats of nuclear pore proteins form a three-dimensional meshwork with hydrogel-like properties. Science.

[23] Reichheld SE, Muiznieks LD, Keeley FW, Sharpe S (2017). Direct observation of structure and dynamics during phase separation of an elastomeric protein. Proc. Natl Acad. Sci. USA.

[24] Murray DT (2017). Structure of FUS protein fibrils and its relevance to self-assembly and phase separation of low-complexity domains. Cell.

[25] Conicella AE, Zerze GHGH, Mittal J, Fawzi NL (2016). ALS mutations disrupt phase separation mediated by α-helical structure in the TDP-43 low-complexity C-terminal domain. Structure.

[26] Shin Y, Brangwynne CP (2017). Liquid phase condensation in cell physiology and disease. Science.

[27] Muiznieks LD, Keeley FW (2017). Biomechanical design of elastic protein biomaterials: a balance of protein structure and conformational disorder. ACS Biomater. Sci. Eng..

[28] Wise SG, Weiss AS (2009). Tropoelastin. Int. J. Biochem. Cell Biol..

[29] Qin G, Hu X, Cebe P, Kaplan DL (2012). Mechanism of resilin elasticity. Nat. Commun..

[30] Cao Q, Wang Y, Bayley H (2004). Sequence of abductin, the molluscan ‘rubber’ protein. Curr. Biol..

[31] Hayashi CY (2002). Molecular architecture and evolution of a modular spider silk protein gene. Science.

[32] Cai H (2017). Self-coacervation of modular squid beak proteins—a comparative study. Soft Matter.

[33] Brady JP (2017). Structural and hydrodynamic properties of an intrinsically disordered region of a germ cell-specific protein on phase separation. Proc. Natl Acad. Sci. USA.

[34] Burke KA, Janke AM, Rhine CL, Fawzi NL (2015). Residue-by-residue view of in vitro FUS granules that bind the C-terminal domain of RNA polymerase II. Mol. Cell.

[35] Pelton JG, Torchia DA, Meadow ND, Roseman S (1993). Tautomeric states of the active‐site histidines of phosphorylated and unphosphorylated IIIGlc, a signal‐transducing protein from escherichia coli, using two‐dimensional heteronuclear NMR techniques. Protein Sci..

[36] Li S, Hong M (2011). Protonation, tautomerization, and rotameric structure of histidine: A comprehensive study by magic-angle-spinning solid-state NMR. J. Am. Chem. Soc..

[37] Mohammadi, P. et al. Phase transitions as intermediate steps in the formation of molecularly engineered protein fibers. *Commun. Biol*. **1**, 86 (2018). 10.1038/s42003-018-0090-y PMC6123624 30271967

[38] Mohammadi, P., Beaune, G., Stokke, B. T., Timonen, J. V. I. & Linder, M. B. Self-coacervation of a silk-like protein and its use as an adhesive for cellulosic materials. *ACS Macro Lett*. **7**, 1120–1125 (2018). 10.1021/acsmacrolett.8b00527 PMC6150716 30258700

[39] Miserez A, Li Y, Waite JH, Zok F (2007). Jumbo squid beaks: inspiration for design of robust organic composites. Acta Biomater..

[40] Miserez A, Rubin D, Waite JH (2010). Cross-linking chemistry of squid beak. J. Biol. Chem..

[41] Ackermann, B. E. & Debelouchina, G. T. Heterochromatin protein HP1α gelation dynamics revealed by solid-state NMR spectroscopy. *Angew. Chemie Int. Ed.* **58**, 6300–6305 (2019). 10.1002/anie.201901141 PMC6482055 30845353

[42] St George-Hyslop P (2018). The physiological and pathological biophysics of phase separation and gelation of RNA binding proteins in amyotrophic lateral sclerosis and fronto-temporal lobar degeneration. Brain Res..

[43] Kroschwald S (2018). Different material states of Pub1 condensates define distinct modes of stress adaptation and recovery. Cell Rep..

[44] Xiang S (2015). The LC domain of hnRNPA2 adopts similar conformations in hydrogel polymers, liquid-like droplets, and nuclei. Cell.

[45] Kato M (2012). Cell-free formation of RNA granules: low complexity sequence domains form dynamic fibers within hydrogels. Cell.

[46] Kim S (2017). Salt triggers the simple coacervation of an underwater adhesive when cations meet aromatic π electrons in seawater. ACS Nano.

[47] Kim S (2016). Complexation and coacervation of like-charged polyelectrolytes inspired by mussels. Proc. Natl Acad. Sci. USA.

[48] Blocher, W. C. & Perry, S. L. Complex coacervate-based materials for biomedicine. *Wiley Interdiscip. Rev. Nanomed.**Nanobiotechnol.* **9**, e1442 (2017). 10.1002/wnan.1442 27813275

[49] Johnson NR, Wang Y (2014). Coacervate delivery systems for proteins and small molecule drugs. Expert Opin. Drug Deliv..

[50] Chu H, Gao J, Chen C-W, Huard J, Wang Y (2011). Injectable fibroblast growth factor-2 coacervate for persistent angiogenesis. Proc. Natl Acad. Sci. USA.

[51] Lim ZW, Ping Y, Miserez A (2018). Glucose-responsive peptide coacervates with high encapsulation efficiency for controlled release of insulin. Bioconjug. Chem..

[52] Roberts S, Dzuricky M, Chilkoti A (2015). Elastin-like polypeptides as models of intrinsically disordered proteins. FEBS Lett..

[53] Quiroz FG, Chilkoti A (2015). Sequence heuristics to encode phase behaviour in intrinsically disordered protein polymers. Nat. Mater..

[54] Solyom Z (2013). BEST-TROSY experiments for time-efficient sequential resonance assignment of large disordered proteins. J. Biomol. NMR.

[55] Orekhov VY, Jaravine VA (2011). Analysis of non-uniformly sampled spectra with multi-dimensional decomposition. Prog. Nucl. Magn. Reson. Spectrosc..

[56] Schanda P, Brutscher B (2005). Very fast two-dimensional NMR spectroscopy for real-time investigation of dynamic events in proteins on the time scale of seconds. J. Am. Chem. Soc..

[57] Van Geet AL (1968). Calibration of the methanol and glycol nuclear magnetic resonance thermometers with a static thermistor probe. Anal. Chem..

[58] Morcombe CR, Zilm KW (2003). Chemical shift referencing in MAS solid state NMR. J. Magn. Reson..

