# Supplementary material for: Hydrogen bond guidance and aromatic stacking drive liquid-liquid phase separation of intrinsically disordered histidine-rich peptides
Source: Nat Commun. 2019 Nov 29;10:5465. doi: 10.1038/s41467-019-13469-8 (PMC6884462; doi:10.1038/s41467-019-13469-8)
Supplement: Supplementary file 1 — Supplementary Information [file 41467_2019_13469_MOESM1_ESM.pdf]

Supplementary Information for manuscript

Hydrogen Bond Guidance and Aromatic Stacking Drive Liquid-Liquid Phase Separation of Intrinsically Disordered Histidine-Rich Peptides

By Gabryelczyk *et al.*

Supplementary Figures

```
        10         20         30         40         50         60
MQLYGAPAVG GVVENAVNAA ESGAAATHDA QGAYAEADTA GVLDVNHAEH HDGVHDASGY

        70         80         90        100        110        120
GFGGLAGHGG FAGHGLYGPG FAGHGLLGAG YAGLGLHGAG FAGHGLHGAG FAGHGLYGAG

       130        140        150        160
FAGHGLHGFA GHGLYGAGFA GHGLGLGGLH GALGHGALAH Y
```

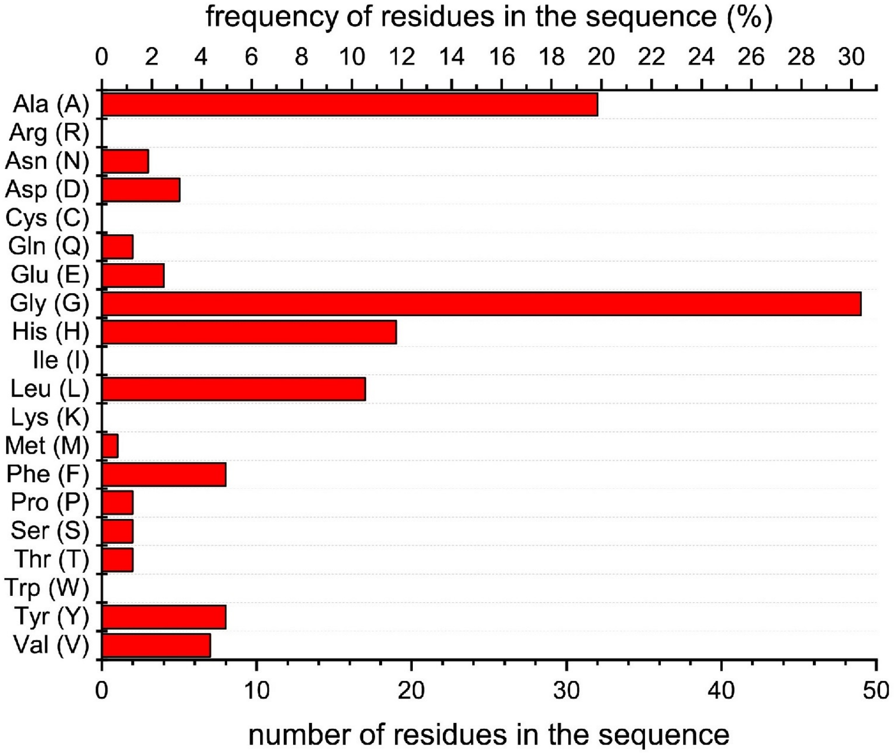

Supplementary Figure 1. Recombinant HBP-1 – amino acid sequence and composition. HBP-1 possesses sequence features characteristic for IDP exhibiting LLPS properties. It has low sequence complexity that lacks Cys, Ile, Lys, Met (added as a starting amino acid in recombinant form of the protein), Arg, and Trp residues. The N-terminus contains negatively-charged residues (Glu, Asp) distributed along His-rich and Ala-rich clusters. The C-terminus lacks acidic residues and contains region of hydrophobic modular penta-repeats (marked in shades of blue and red) that are enriched mainly with Ala, Gly, His, Leu, Phe and Tyr residues. Source data are provided as a Source Data file.

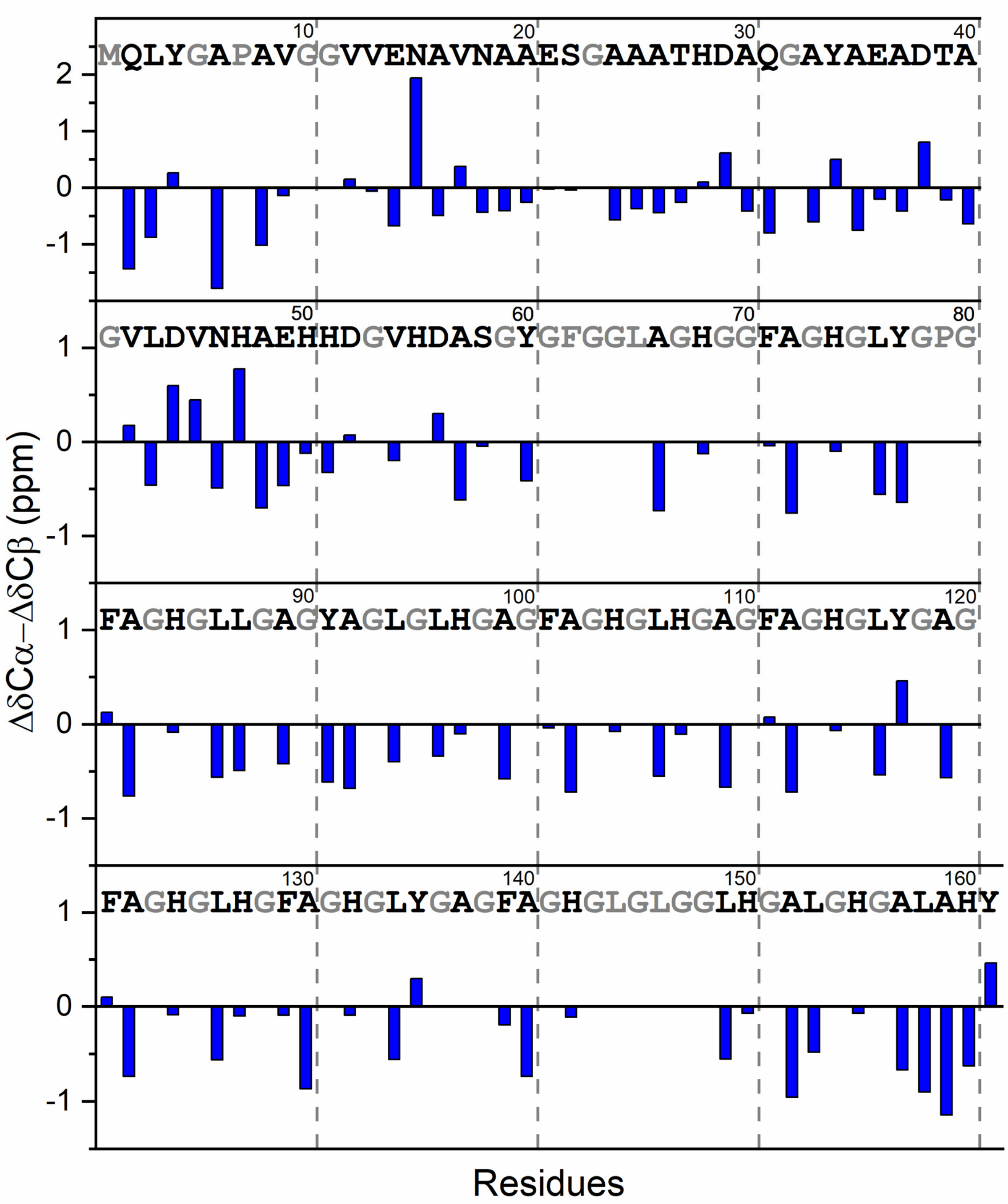

Supplementary Figure 2. Residue-specific secondary structure propensity of HBP-1. Secondary chemical shifts ($\Delta\delta C_{\alpha}$-$\Delta\delta C_{\beta}$) indicate lack of secondary structure formation. Calculation not performed for unassigned residues and glycine, both marked in grey. Values on the x-axis represent residue numbers. Source data are provided as a Source Data file.

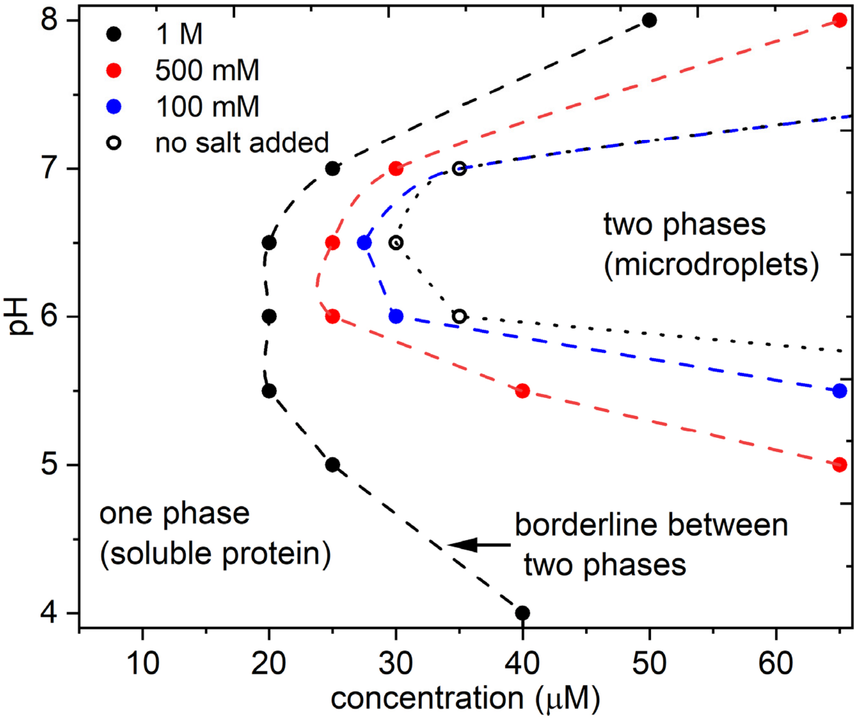

Supplementary Figure 3. Phase diagrams of HBP-1. Different pH values as a function of protein concentration and ionic strength. The estimated boundary lines determine the pH at which LLPS (micro-droplets) was first observed. Ionic strength of the buffers (Supplementary Table 1) adjusted with NaCl. Source data are provided as a Source Data file.

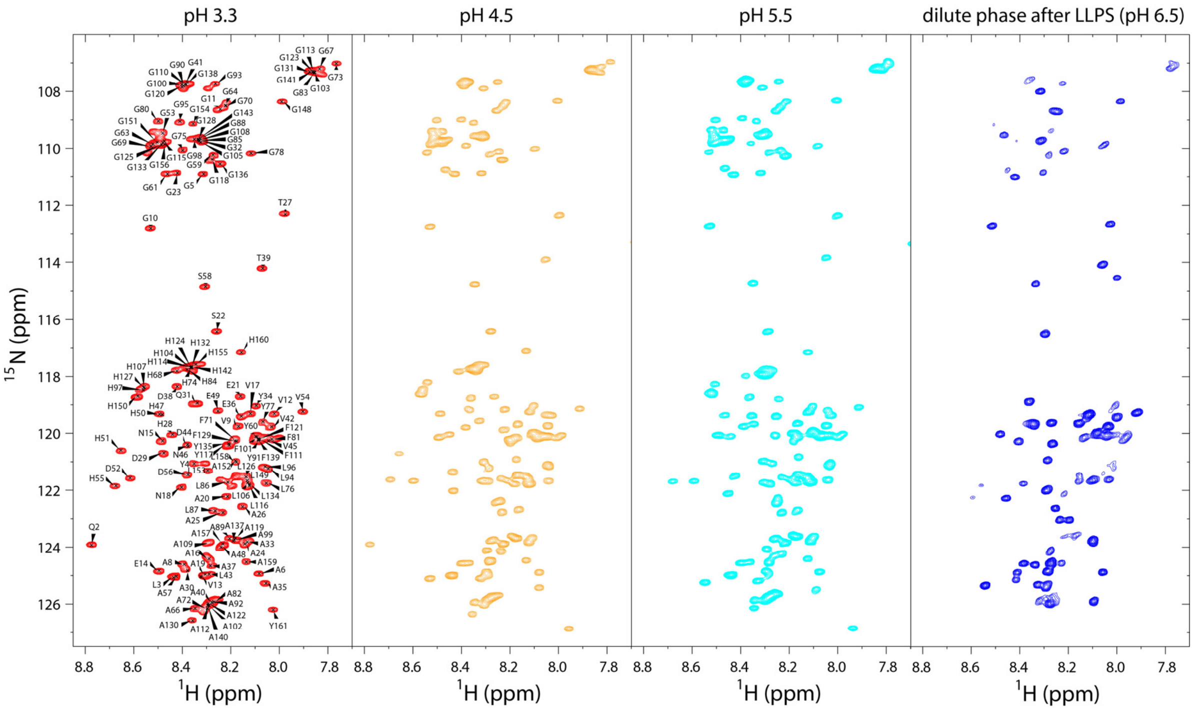

Supplementary Figure 4. ^{1}H-^{15}N-HSQC spectra of HBP-1 at different pH values. Spectra acquired at T = 298 °K and a protein concentration of 2 mg·mL^{-1} (130 μM).

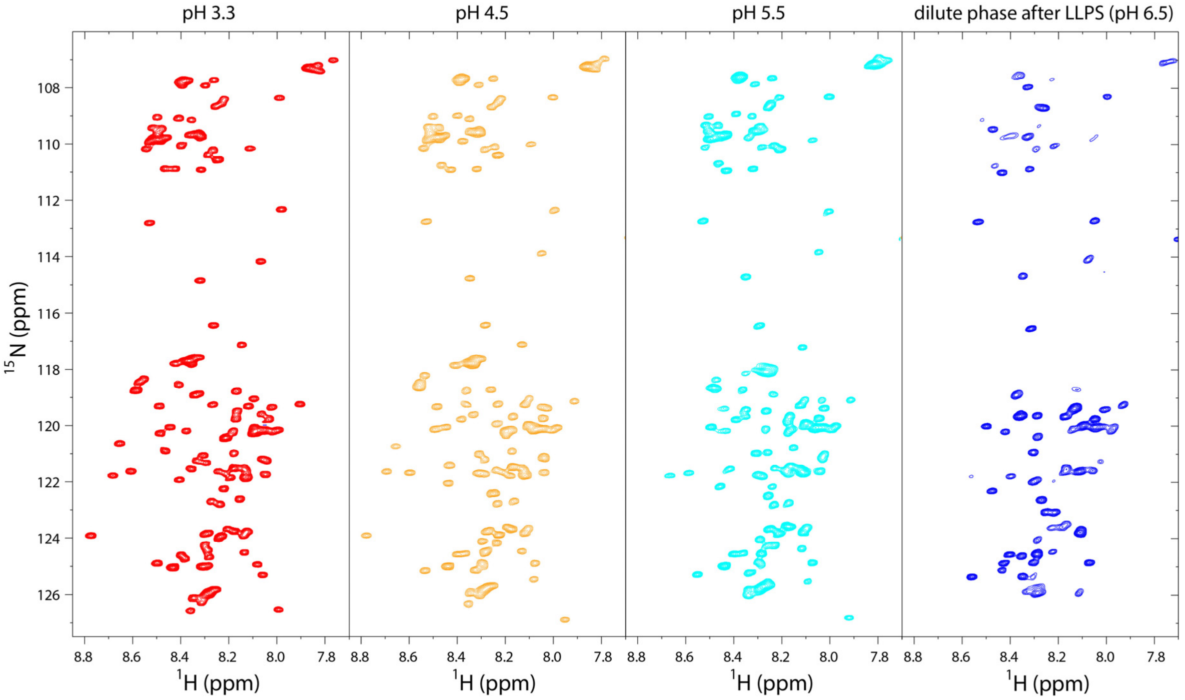

Supplementary Figure 5. ^{1}H-^{15}N-HSQC spectra of HBP-1 at various pH values at low protein concentration. Spectra acquired at a protein concentration of 0.5 mg·mL^{-1} (32 µM) *i.e.* 75 % lower concentration compared to the initial conditions (Supplementary Figure 4), T = 298 °K.

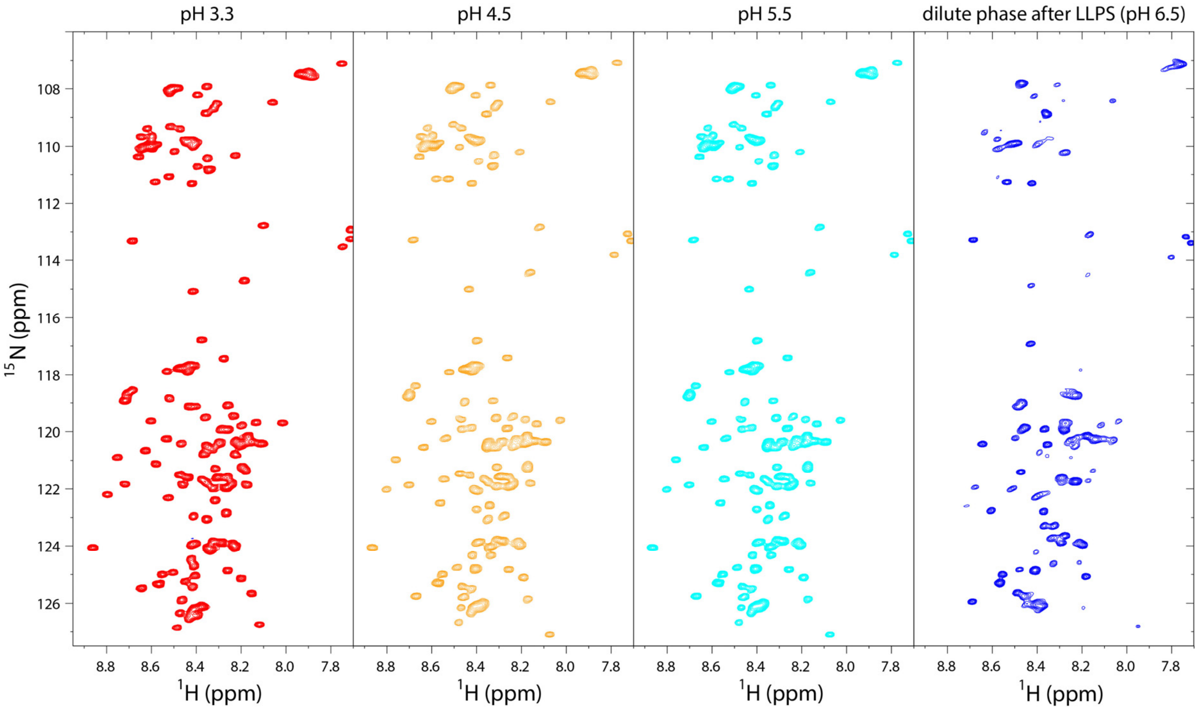

Supplementary Figure 6. ^{1}H-^{15}N-HSQC spectra of HBP-1 at various pH values at low temperature. Spectra acquired at T = 279 °K compared to T = 298 °K at initial conditions (Supplementary Figure 4). Protein concentration = 0.5 mg·mL^{-1} (32 µM).

a) **V1-C (6xHis-TEV-G67 – Y161)**
HMHHHHHHSSGVDLGTENLYFQSMMGHGGFAGHGLYGPGFAGHGLLGAGYAGLGLHGAGFAGHGLHGAG
FAGHGLYGAGFAGHGLHGFAGHGLYGAGFAGHGLGLGGLHGALGHGALAHY

V2-C (6xHis-TEV-G98 – Y161)
HMHHHHHHSSGVDLGTENLYFQSMMGAGFAGHGLHGAGFAGHGLYGAGFAGHGLHGFAGHGLYGAGFAG
HGLGLGGLHGALGHGALAHY

b) **V3-N(M1 - A102)**
MQLYGAPAVGGVVENAVNAAESGAAATHDAQGAYAEADTAGVLDVNHAEHHDGVHDASGYGFGGLAGHG
GFAGHGLYGPGFAGHGLLGAGYAGLGLHGAGFAK

V4-N(M1 - H107)
MQLYGAPAVGGVVENAVNAAESGAAATHDAQGAYAEADTAGVLDVNHAEHHDGVHDASGYGFGGLAGHG
GFAGHGLYGPGFAGHGLLGAGYAGLGLHGAGFAGHGLHK

V5-N(M1 - A112)
MQLYGAPAVGGVVENAVNAAESGAAATHDAQGAYAEADTAGVLDVNHAEHHDGVHDASGYGFGGLAGHG
GFAGHGLYGPGFAGHGLLGAGYAGLGLHGAGFAGHGLHGAGFAK

V6-N(M1 - Y117)
MQLYGAPAVGGVVENAVNAAESGAAATHDAQGAYAEADTAGVLDVNHAEHHDGVHDASGYGFGGLAGHG
GFAGHGLYGPGFAGHGLLGAGYAGLGLHGAGFAGHGLHGAGFAGHGLYK

V7-N(M1 - H122)
MQLYGAPAVGGVVENAVNAAESGAAATHDAQGAYAEADTAGVLDVNHAEHHDGVHDASGYGFGGLAGHG
GFAGHGLYGPGFAGHGLLGAGYAGLGLHGAGFAGHGLHGAGFAGHGLYGAGFAK

V3-C (G103 – Y161)
GHGLHGAGFAGHGLYGAGFAGHGLHGFAGHGLYGAGFAGHGLGLGGLHGALGHGALAHY

V4-C (G108 – Y161)
GAGFAGHGLYGAGFAGHGLHGFAGHGLYGAGFAGHGLGLGGLHGALGHGALAHY

V5-C (G113 – Y161)
GHGLYGAGFAGHGLHGFAGHGLYGAGFAGHGLGLGGLHGALGHGALAHY

V6-C (G118 – Y161)
GAGFAGHGLHGFAGHGLYGAGFAGHGLGLGGLHGALGHGALAHY

V7-C (G123 – Y161)
GHGLHGFAGHGLYGAGFAGHGLGLGGLHGALGHGALAHY

c) **GY-23 (G113 – Y135)**
GHGLYGAGFAGHGLHGFAGHGLY

GA-25 (G98 – A122)
GAGFAGHGLHGAGFAGHGLYGAGFA

GH-25 (G103 – H127)
GHGLHGAGFAGHGLYGAGFAGHGLH

Supplementary Figure 7. Sequences of HBP-1 protein variants. (a) V1-C and V-2C (were expressed with His-tag and TEV protease recognition site). We attempted to carry out proteolytic cleavage of the His-tag; however we could not achieve efficient cleavage since the optimum pH for the TEV protease was within the coacervation range of the variants. We observed that the variants containing the 6xHis-TEV tag underwent LLPS in the same way as full-length protein. Thus, we concluded that the tag did not have any effect on their LLPS properties. **(b)** Variants obtained from trypsin cleavage of the protein mutants containing extra Lys residue. The mutants were created by genetically introducing a single Lys residue into the protein sequence that served as a recognition site for trypsin cleavage. Since the wild type HBP-1 sequence completely lacks Arg and Lys, the addition of an extra Lys residue allowed introducing a single specific cleavage site that could be precisely recognized by trypsin cleaving the protein into two fragments (referred here as N- and C- terminus fragments, e.g. Vx-N and Vx-C, respectively). **(c)** Synthetic peptides with modular repeats.

a) **HBP-2 (M1 - L175)**
MQFFGAGPFNTAHHSAVSDAAAAHHDAAGEYAQNAATGLLDTHHNENHDMTHDLANGYGLHEHDEQHHGL
ADGLHQEYAA**R**AAQGANAVHNDAAQSHSALAAANTFGHGHAPYAAY**GHGVYGHGPYGHGPYGHGLYGHGL**
YGHGPYGHGLYGHGAFGHGLNAYAPLVGHGL**R**GYL

b) **HBP-2-N (M1 - R81)**
MQFFGAGPFNTAHHSAVSDAAAAHHDAAGEYAQNAATGLLDTHHNENHDMTHDLANGYGLHEHDEQHHGL
ADGLHQEYAA**R**

HBP-2-C (A82 - R172)
AAQGANAVHNDAAQSHSALAAANTFGHGHAPYAAY**GHGVYGHGPYGHGPYGHGLYGHGLYGHGPYGHGLY**
GHGAFGHGLNAYAPLVGHGL**R**

c)
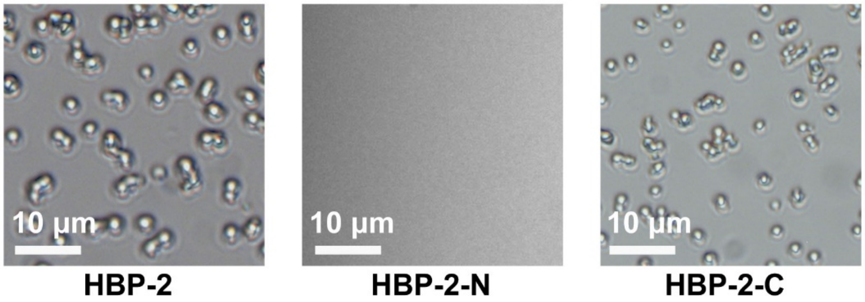

Supplementary Figure 8. Recombinant HBP-2. (a) Amino acid sequence of the protein with indicated trypsin sites (R in bold font) and GHGxY motif (green) **(b)** HBP-2-N- and -C- terminal fragments obtained after trypsin cleavage. **(c)** Optical micrographs after LLPS. Protein concentration 2 mg·mL^{-1}.

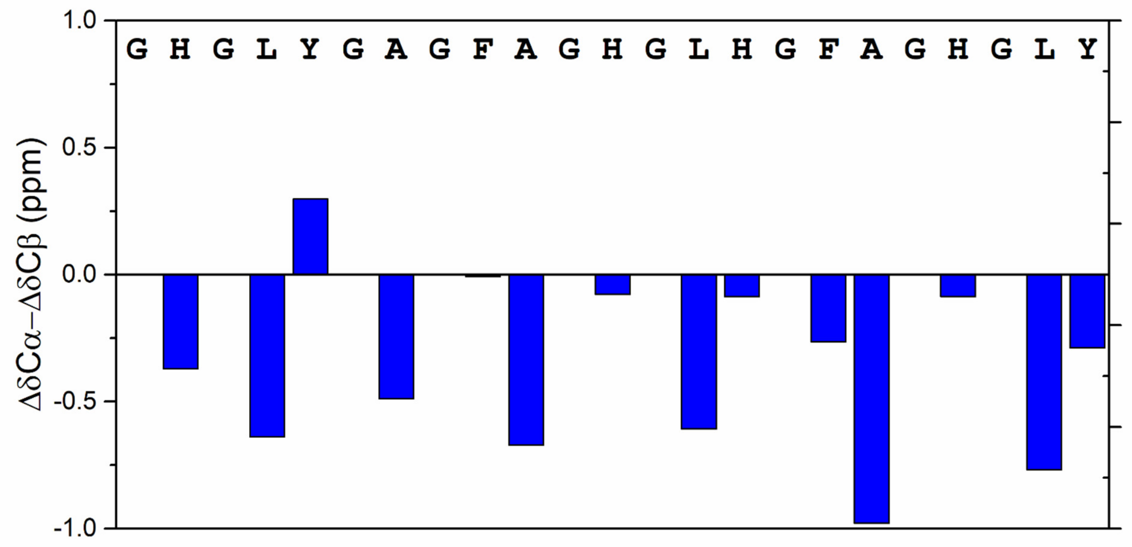

Supplementary Figure 9. Residue-specific secondary structure propensity of GY-23 peptide. Secondary shifts ($\Delta\delta C_\alpha$-$\Delta\delta C_\beta$) indicate lack of secondary structure formation. Source data are provided as a Source Data file.

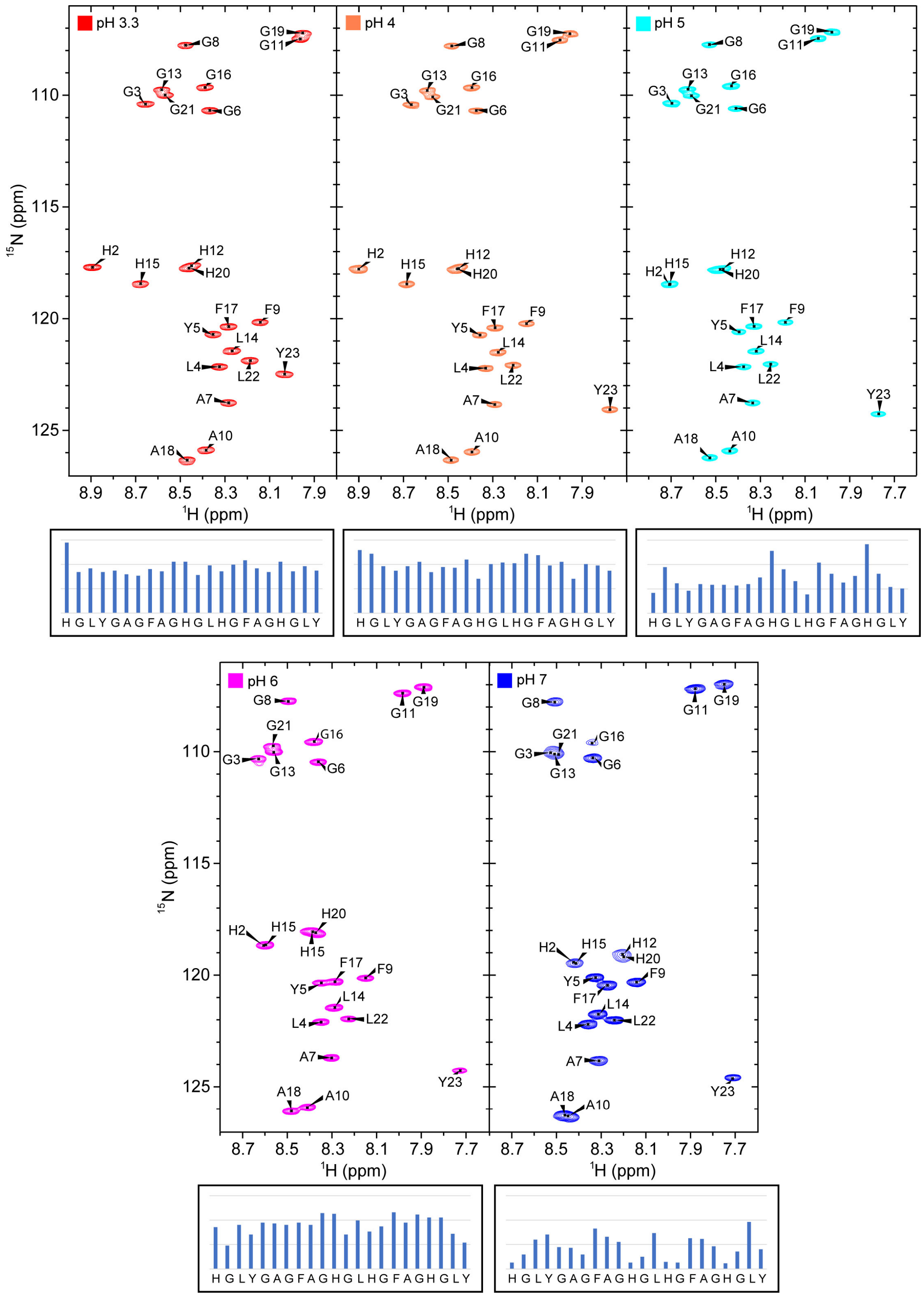

Supplementary Figure 10. ^{1}H-^{15}N-HMQC spectra of GY-23 at different pH values. Bar graphs represent relative peak intensity for the assigned residues. Spectra acquired at 298 °K and a peptide concentration of 1.5 mM. Source data are provided as a Source Data file.

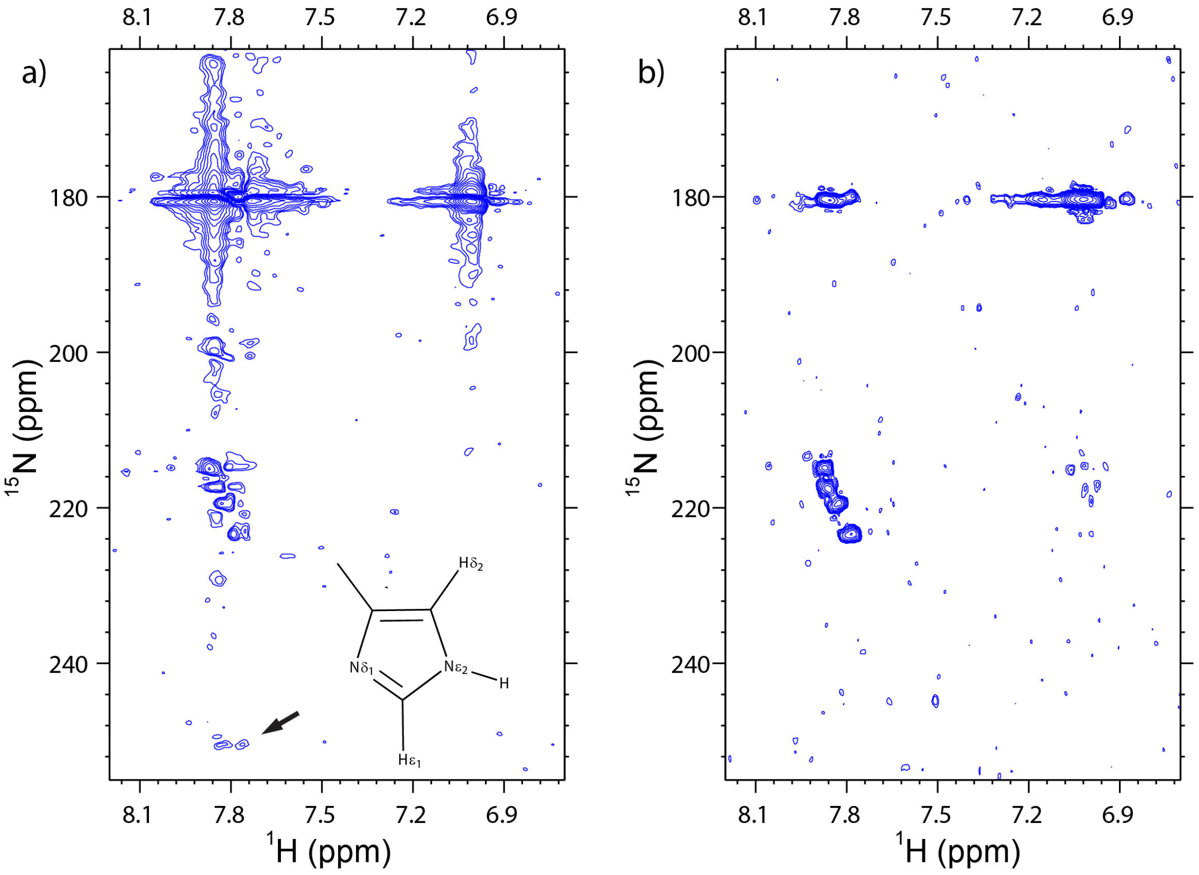

Supplementary Figure 11. Long-range ^{1}H-^{15}N-HMQC spectra of His imidazole ring. Time-dependent comparison of **(a)** the spectrum acquired within 5 min after pH adjustment to 7.0 (point of coacervation) and **(b)** the spectrum acquired within 30 min after pH adjustment. The characteristic resonance for ε-tautomer at approximately 250 ppm in the N-dimension (marked with the arrow in panel a) is present in the time frame of a few minutes shortly after pH adjustment to the coacervation point, indicating the transient nature of the interaction. Spectra acquired at T = 298 °K and a peptide concentration of 1.5 mM.

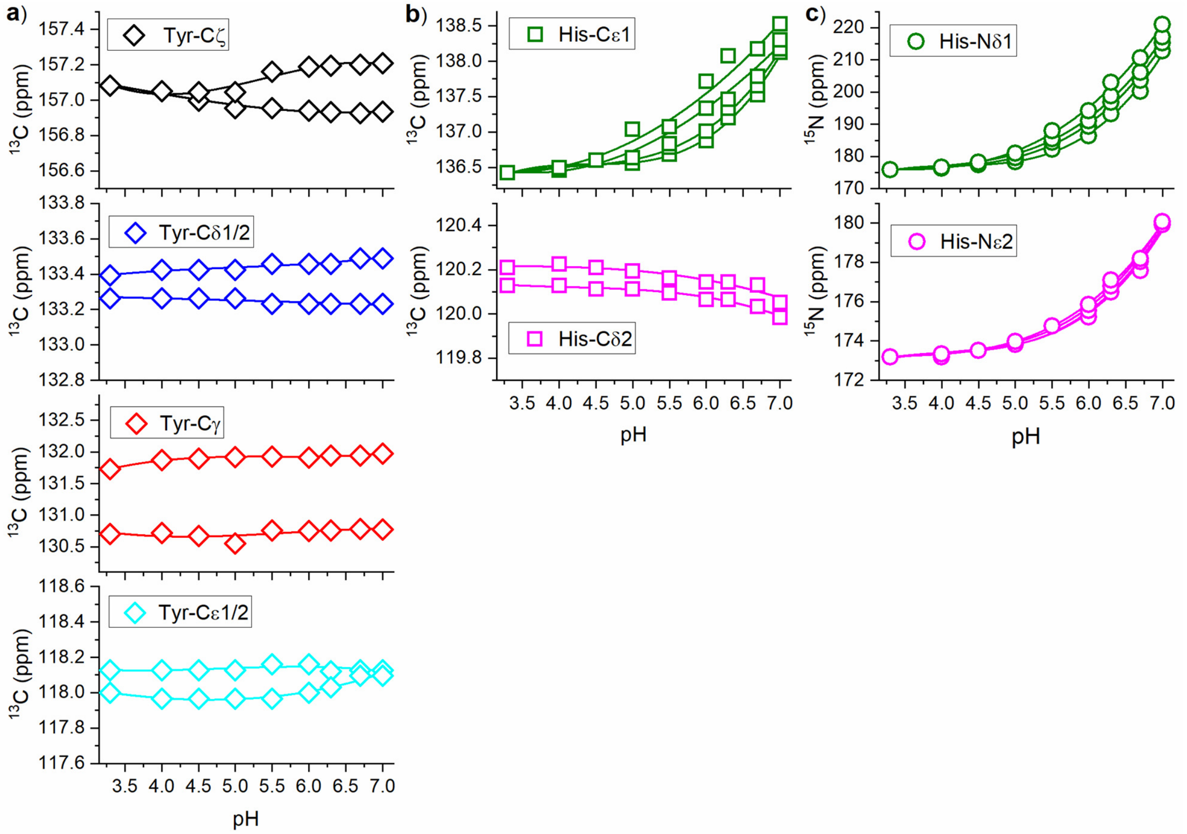

Supplementary Figure 12. Chemical shifts trajectories of ^{13}C atoms of Tyr as well as ^{13}C and ^{15}N of His. Chemical shifts at different pH values: **(a)** ^{13}C atoms of Tyr aromatic ring, **(b)** ^{13}C and **(c)** ^{15}N atoms of His imidazole side chain. Fitted curves (as a guide for eye) indicate chemical shift trajectories. Chemical shift of one Tyr-C_{ζ} spins (the apical carbon in the aromatic ring), His-$C_{\varepsilon 1}$, and $N_{\delta 1}$ spins show very similar trajectories indicating that they are involved in the interaction. Note the split of chemical shifts for Tyr-C_{ζ} above pH ~ 5. The remaining chemical shifts are less affected by pH changes. Source data are provided as a Source Data file.

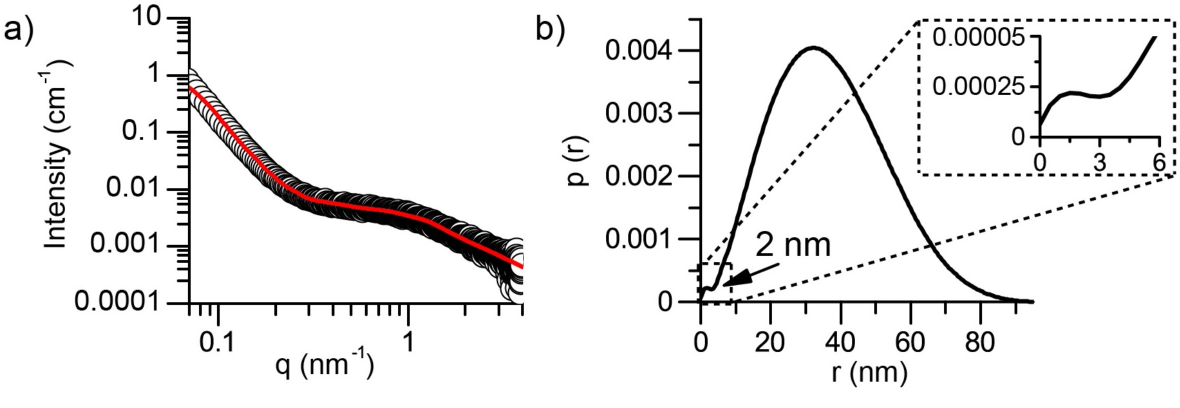

Supplementary Figure 13. Additional SAXS experiments with a synchrotron X-ray source. (a) SAXS experimental curve. The calculated fit for the peptide assemblies from the IFT method is presented as the full red line. **(b)** Corresponding $p(r)$ profile calculated from the SAXS data in (a) using Supplementary Equation 2. The arrow and the zoom-in view (inset) indicates that coacervate micro-droplets contain self-assembled sub-units of *ca*. 2 nm, which may be attributed to peptide oligomeric units. Source data are provided as a Source Data file.

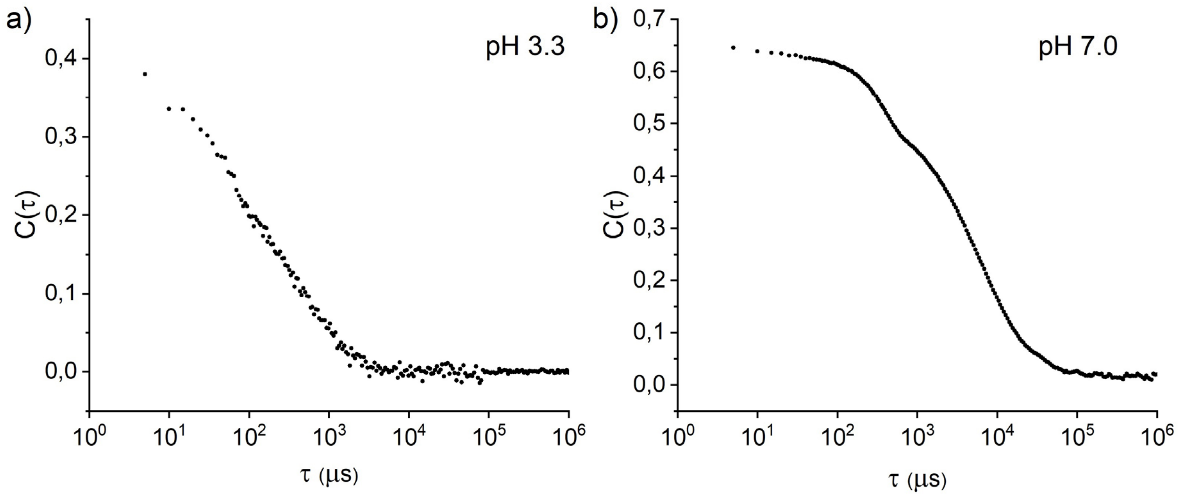

Supplementary Figure 14. Correlation functions of the DLS measurements of GY-23 peptide. Plots showing the intensity correlation function C(τ) *vs.* the correlation time (τ) before and after LLPS (Figure 5c) **(a)** Sample before LLPS (10 mM in acetic acid, pH 3.3). The curve with a single decay at short correlation times represents a monomodal distribution of rather small particles in the nanometer range. **(b)** Sample after LLPS (coacervation buffer, pH 7.0). The correlation function shows two decays at higher correlation times compared to (a) indicating the presence of two distinct populations of larger particles (coacervates). Source data are provided as a Source Data file.

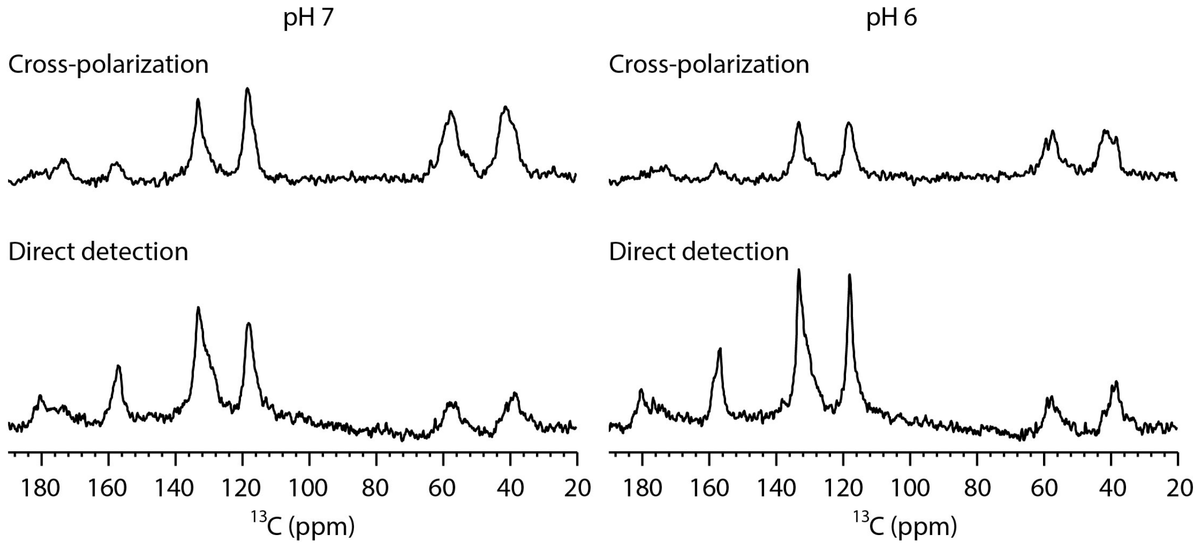

Supplementary Figure 15. Analysis of molecular mobility of tyrosine residues in GY-23 peptide. Comparison of ^{1}H-^{13}C cross-polarization (CP) with ^{13}C detection and direct ^{13}C detection 1D solid-state NMR spectra of Tyr 5 and Tyr 23 labelled GY-23 peptide at pH 6 and 7. The increased mobility of Tyr residues at pH 6 is indicated by the observed lower signal intensity of the CP spectrum, and by the sharper peaks in the directly detected carbon spectrum (with integrals consistent with the spectrum recorded at pH 7), respectively.

Supplementary Tables

Supplementary Table 1. List of buffers used in LLPS studies of HBP-1 protein, its variants, and HBP peptides at different pH and ionic strength.

pH range	Buffer (50 mM)
5.0 – 5.5	Sodium acetate
6.0 – 7.0	Sodium phosphate
7.5 – 9.0	Tris-HCl

Each buffer was prepared at 0.1 M, 0.5 M, and 1 M ionic strength (adjusted with NaCl). Ionic strength defined as the sum of molar concentrations of a salt component of a buffer and NaCl.

Supplementary Methods

Protein expression and purification

Genetic constructs encoding wild type HBP-1 and -2 proteins were obtained from DNA2.0/ATUM (USA), HBP1- V1-C and -V2-C variants were provided by Protein Production Platform (School of Biological Sciences, NTU, Singapore), HBP-1-V-3-7 were purchased from GenScript (USA). The proteins and their variants were expressed in *E. coli* and purified with reverse-phase HPLC using the protocols developed for the wild type HBP-1 and -2 proteins, described previously[1,2]. Briefly, BL-21 *E. coli* strain was transformed with plasmids containing target protein genes. Bacteria were then cultivated at 37 °C until OD_{600} = 0.7 – 0.8 in LB medium supplemented with appropriate antibiotic. Protein expression was induced with 0.5 mM IPTG (Isopropyl β-D-1-thiogalactopyranoside) and carried out for 4 – 5 h with 220 RPM shaking at 37 °C. Bacterial cells were harvested by centrifugation at 10000 *g* for 15 min at 4 °C. Cell pellets were frozen at -20 °C and then resuspended in a lysis buffer (50 mM Tris-HCl pH 8.0, supplemented with SIGMAFAST protease inhibitor cocktail). Cell lysis was performed with a microfulidizer (18000 psi, 5 passes, 4 °C). Cell lysate was mixed with ice cold 100 % acetic acid in a volume ratio of 1:20 (acetic acid/cell lysate), homogenized by gentle vortexing, and centrifuged (38900 *g*, 30 min, 4 °C). The pellet (containing precipitated impurities) was discarded while the supernatant (containing target protein) was subjected to reverse-phase HPLC system equipped with Kromasil RP-300-C8 column (AkzoNobel , Sweden). Proteins were eluted from the column with a linear gradient of acetonitrile (ACN) containing 0.1 % trifluoroacetic acid (TFA). Purified products were freeze-dried to remove the solvents.

Trypsin cleavage

The lyophilized HBP-1-V3-7 variants and HBP-2 (wild type) were dissolved in 50 mM Tris-HCl buffer (pH 8.8) to the final protein concentration of 1 $mg \cdot mL^{-1}$. Trypsin powder (Trypsin Gold, MS grade, Promega) was dissolved in 50 mM acetic acid to the final concentration of 1 $mg \cdot mL^{-1}$. Enzymatic cleavage was carried out at 37 °C for 2 h at a 1/1000 trypsin/protein volume ratio. The reaction was terminated with 1 mM (final concentration) PMSF (phenylmethylsulfonyl fluoride). Products of the enzymatic cleavage were purified with reverse-phase HPLC using a Kromasil RP-300-C8

column (AkzoNobel, Sweden) and a linear gradient of ACN containing 0.1 % TFA. The purity of separated N- and C-terminal variants was assessed by SDS-PAGE and their molecular weight verified by MALDI-TOF mass spectrometry using an AXIMA Performance MALDI TOF/TOF Mass Spectrometer (Shimadzu Biotech). Purified products of enzymatic cleavage were freeze-dried and re-solubilized in an appropriate buffer for further studies.

Synthetic peptides

All unlabeled peptides were synthesized and purified by HPLC (final purity > 95 %) by GL Biochem Ltd (China). GY-23 peptide with ^{13}C and ^{15}N labelled tyrosine residues was synthesized and purified using HPLC (final purity > 90 %) by GenScript (USA).

Isotope labelling of NMR samples

^{13}C- and ^{15}N-labeled HBP-1 protein was expressed in *E. coli* (BL-21 (DE3) strain). A plasmid carrying the protein gene was transformed into chemically competent cells. Bacteria were cultivated in M9 minimal medium containing ^{13}C-glucose (2.5 $g \cdot L^{-1}$) and ^{15}N-ammonium chloride (1 $g \cdot L^{-1}$), supplemented with biotin (1 $mg \cdot L^{-1}$) and thiamin (1 $mg \cdot L^{-1}$). Protein expression was induced with 0.5 mM IPTG when OD_{600} reached 0.6. The induced bacterial culture was incubated for 8 h at 37 °C at 225 RPM and harvested by centrifugation (3900 *g*, 15 min, 4 °C). Cell lysis and protein purification was carried out as described above.

Uniformly ^{13}C and ^{15}N labelled GY-23 peptide was expressed using modified gene of HBP-1-V5 protein variant. The construct was composed of the N-terminal part (M1-A112) of the HBP-1 protein, followed by a trypsin recognition site (K), the GY-23 sequence, and a stop codon. The fusion protein was expressed and purified using the same protocol as for ^{13}C- ^{15}N-HBP-1 protein. The GY-23 peptide was cleaved off from HBP-1(M1-A112) tag with trypsin and purified as described above.

Additional SAXS Data

SAXS measurements were performed at the cSAXS beamline at PSI (Viligen, Switzerland). After mixing the peptides with coacervation buffer, the coacervates were equilibrated for at least 1 h and sealed in thin-walled quartz capillaries for SAXS measurements. An X-ray beam with a

wavelength of 1.11 Å (11.2 keV) was used, with a sample to detector distance of 2152 mm providing $0.05 < q < 5$ nm^{-1}, where q is the length of the scattering vector, defined by Supplementary Equation 1:

$$q = \frac{4\pi}{\lambda \sin\left(\frac{\theta}{2}\right)} \qquad (1)$$

λ being the wavelength and ϑ the scattering angle. The 2D SAXS patterns were acquired at 12 positions on each capillary in triplets for 1 s (36 measurements per sample) using a Pilatus 2M detector (Dectris Ltd, Baden, Switzerland; active area 254 x 289 mm^2 with a pixel size of 172 x 172 µm^2) and integrated into the one-dimensional scattering function I(q) after inspection for beam damage. No beam damage was observed in all investigated samples. The scattering curves were plotted as a function of intensity, I versus q. Scattering from the corresponding buffer was subtracted as background from all samples before further analysis.

SAXS Data Analysis

The p(r) was calculated from the scattered intensity I(q) using the Supplementary Equation 2[3]:

$$\mathrm{I}(q) = 4\pi \int_0^{\infty} \mathrm{p}(r) \frac{\sin(qr)}{qr} \mathrm{d}r \qquad (2)$$

and gives a real space representation of the overall shape of the particles. The scattering of mass fractal like aggregates was calculated according to Mildner *et al* [4] using SasView.

Prediction of isoelectric point of HBP-1 protein

Isoelectric point (pI) of the HBP-1 protein was predicted using ProtParam tool available online: https://web.expasy.org/protparam/.

Calculation of secondary shifts

Secondary chemical shifts ($\Delta\delta$) for C_α and C_β were calculated using the Supplementary Equation 3:

$$\Delta\delta = \delta_{obs} - \delta_{rc} \qquad (3)$$

were δ_{obs} is the observed chemical shift and δ_{rc} is the random coil chemical shift. Random coil reference values obtained from BMRB chemical shift statistics database

(http://www.bmrb.wisc.edu/ref_info/statful.htm). The data were presented as the difference $\Delta\delta C_{\alpha}$ - $\Delta\delta C_{\beta}$.

Supplementary References

1. Cai, H. *et al.* Self-coacervation of modular squid beak proteins – a comparative study. *Soft Matter* **13**, 7740–7752 (2017).
2. Tan, Y. *et al.* Infiltration of chitin by protein coacervates defines the squid beak mechanical gradient. *Nat. Chem. Biol.* **11**, 488–495 (2015).
3. Glatter, O. A new method for the evaluation of small-angle scattering data. *J. Appl. Crystallogr.* **10**, 415–421 (1977).
4. Meakin, P., Roldughin, V. I., Roldughin, V. I. & Allen, A. Small-angle scattering from porous solids with fractal geometry. *J. Phys. D: Appl. Phys*. **19** 1535–1545 (1986).
